# Discovery of novel sulphonamide hybrids that inhibit LSD1 against bladder cancer cells

**DOI:** 10.1080/14756366.2021.2014830

**Published:** 2022-03-30

**Authors:** Jia Liu, Xingwang Zhu, Liu Yu, Minghuan Mao

**Affiliations:** Department of Urology, The 4th affiliated hospital of China Medical University, Shenyang, PR China

**Keywords:** Bladder cancer, sulphonamide, LSD1, HT1376 cells

## Abstract

**Aim:**A series of sulphonamide hybrids were designed, synthesised, and identified as potential lysine-specific demethylase 1 (LSD1) inhibitors.

**Materials and methods:** Bladder cancer cell lines were cultured to evaluate the antiproliferative activity. Inhibitory evaluation of sulphonamide hybrids against LSD1 were performed.

**Conclusion:** sulphonamide derivative **L8** exhibited the antiproliferative activity against HTB5, HTB3, HT1376, and HTB1 cells with IC_50_ values of 1.87, 0.18, 0.09, and 0.93 μM, respectively. Compound **L8** as a selective and reversible LSD1 inhibitor could inhibit LSD1 with the IC_50_ value of 60 nM. It effectively inhibited LSD1 by increasing the expression levels of H3K4me1, H3K4me2, and H3K9me2 in HT1376 cells. To the best of our knowledge, this was the first report which showed that sulphonamide–quinoline–dithiocarbamate hybrids potently inhibited LSD1 in bladder cancer cells. Our studies give the potential application of the sulphonamide-based scaffold for developing LSD1 inhibitors to treat bladder cancer.

## Graphical Abstract

**Figure:**
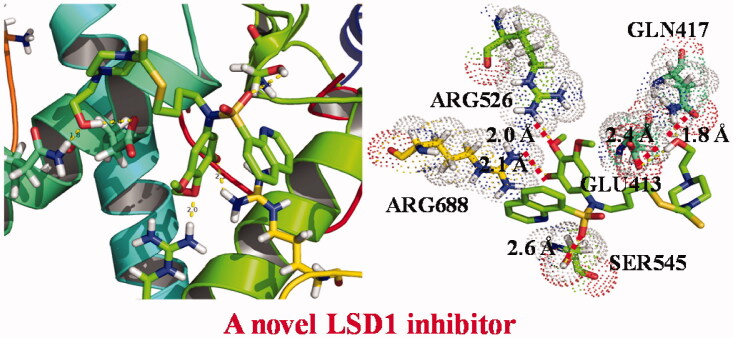

## Graphical abstract

**L8** exhibits the antiproliferative activity against bladder cancer cells.

**L8 i**s a selective and reversible LSD1 inhibitor.

**L8** increases the expression levels of H3K4me1, H3K4me2 and H3K9me2.

## Introduction

Bladder cancer is the most frequently diagnosed malignancy in the urinary system and has the high morbidity and mortality rates^1^. Chemotherapy plays an important role in the treatment of bladder cancer and it is urgent to develop potent anti-bladder cancer drugs^2^^,^^3^. Histone lysine-specific demethylase 1 (LSD1) could catalyse the demethylation of mono and dimethylated H3K4me1/2 or H3K9me1/2 and demethylate many other nonhistone substrates^4^. LSD1 is aberrantly expressed in many malignant tumours such as prostate, ovarian, gastric, liver, breast, lung, bladder, neuroblastoma, and blood cancers^5^. The inhibition of LSD1 could prevent tumour cell proliferation, stimulate antitumor immunity, and enhance antitumor efficacy of immune checkpoint blockade^6^. Therefore, LSD1 has been considered as a potential cancer therapeutic target to discover novel anti-bladder cancer agents^7–9^. LSD1 and MAO-A/-B were belonged to the monoamine oxidase family, and MAO-A/-B shared the similar enzymatic mechanisms and the same cofactor of LSD1 in the cleavage of the inactivated carbon–nitrogen bonds from their substrates^10^. Although a variety of LSD1 inhibitors have been reported to date, many of them show insufficient selectivity towards LSD1^11^.

**Figure 1.**
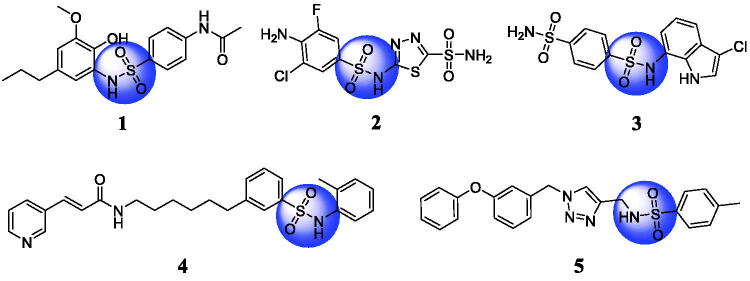
Chemical structures of anticancer sulphonamide derivatives.

Sulphonamide has been proven to be an interesting scaffold and many sulphonamide derivatives are designed as potent antitumor agents for cancer therapy^12^. Phenylpropanoid-based sulphonamide **1** (Figure 1) induced cell cycle arrest at G1/S transition by reducing the expression levels of cyclin D1 and cyclin E in MCF7 cells^13^. Sulphonamide **2** as a potential antitumor agent was a novel tumour-associated isozyme carbonic anhydrase IX inhibitor^14^. Sulphonamide **3** showed a significant antitumor activity against HCT116 human colon carcinoma *in vitro* and *in vivo*^15^. *Trans*-3-(pyridin-3-yl)acrylamide-derived sulphamide **4** showed a single-digit nanomolar antiproliferative activity against DU145, Hela, and H1975 cells and inhibited NAMPT with an IC_50_ value of 5.08 nM^16^. Benzenesulphonamide **5** showed the potent inhibitory effects against PC-3 cells with an IC_50_ value of 4.08 µM as a potential tubulin polymerisation inhibitor^17^.

**Figure 2.**
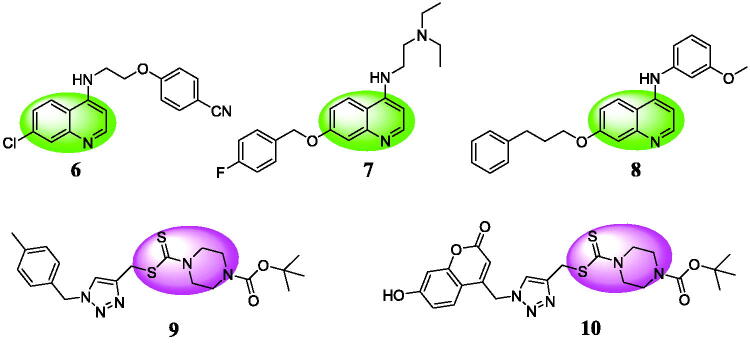
Chemical structures of anticancer quinolines and dithiocarbamate based LSD1 inhibitors.

Quinolines and dithiocarbamates also represent a large group of anticancer agents and there have been many studies on the structural modification based on quinolines or dithiocarbamates^18^^,^^19^. Quinoline **6** (Figure 2) showed the potently inhibitory activity against Raji cells by inducing changes in the cell cycle^20^. Quinoline derivative **7** triggered p53/Bax-dependent apoptosis by activating p53 transcriptional activity against colorectal cancer HCT-116 cells^21^. Quinoline **8** was a potent antiproliferative agent against HCT-116, RKO, A2780, and Hela cell lines with IC_50_ values of 2.56, 3.67, 3.46, and 2.71 μM, respectively^22^. Dithiocarbamate **9** showed the potent cytotoxicity against MGC-803 and HGC-27 cells by the specific and robust inhibition of LSD1^23^. Dithiocarbamate **10** displayed the potent and reversible inhibition against LSD1 with an IC_50_ value of 0.39 μM^24^. So, dithiocarbamate moiety might be a promising fragment to design novel LSD1 inhibitors.

Molecular hybridisation is a useful strategy in anticancer drug design based on the combination of different bioactive scaffolds to produce a new molecular architecture^25^. Based on these findings, we proposed that the sulphonamide derivatives with quinoline and dithiocarbamate groups might have the excellent anticancer activity and LSD1 inhibitory activity. Thus, in this study, we designed a series of sulphonamide–quinoline–dithiocarbamate hybrids *via* a molecular hybridisation strategy and evaluated for their antiproliferative activity and inhibitory activity against LSD1.

## Experimental

### Materials and methods

#### General procedure for the preparation of J1∼J7

A solution of aromatic amine (**11** or **13**, 3.5 mmol) and triethylamine (5.25 mmol) in acetone (10 ml) was cooled to 0 °C. Sulphonyl chloride derivative (**12** or **14**, 5.25 mmol) in acetone (10 ml) was added drop-wise into the solution. After being stirred for 30 min, the reaction mixture was stirred at room temperature for 8 h. The reaction mixture was washed with water (50 ml) and extracted with ethyl acetate (30 ml). The organic extracts were purified by column chromatography on silica (petroleum ether/ethyl acetate = 10/1) to yield compounds **J1∼J7**.

#### General procedure for the preparation of K1∼K3

To a solution of sulphanilamide **J7** (1.2 mmol) and potassium hydroxide (1.5 mmol) in dichloromethane (6 ml) was added 1,2-dibromoethane or 1,3-dibromopropane or 1,4-dibromobutane (1.5 mmol). The reaction mixture was stirred at 60 °C for 10 h. The reaction was diluted with brine (45 ml) and extracted with dichloromethane (25 ml). The organic extracts were purified by column chromatography on silica (petroleum ether/ethyl acetate = 9/1) to yield compounds **K1∼K3**.

#### General procedure for the preparation of L1∼L8

A solution of sulphanilamide intermediates **K1∼K3** (2 mmol), carbon disulphide (3 mmol), trisodium phosphate dodecahydrate (0.5 mmol), and different piperazine derivatives (3 mmol) in acetone (10 ml) was stirred at room temperature overnight. Solvents were removed and crude products were purified by column chromatography on silica (petroleum ether/ethyl acetate = 8/1) to yield compounds **L1∼L8**. The structures of all synthesised compounds **J1∼J7**, **K1∼K3,** and **L1∼L8** were characterised by ^1^H NMR, ^13 ^C NMR, and HRMS. Detailed analytical information was listed in Supporting Information.

#### 3,4-Dimethoxy-N-(quinolin-8-yl)benzenesulphonamide (J1)

White solid, yield:80%, m.p.:1 6 7 ∼ 169 °C. ^1^H NMR (400 MHz, DMSO-d_6_) *δ* 9.84 (s, 1H), 8.88 (dd, *J*= 4.2, 1.6 Hz, 1H), 8.36 (dd, *J* = 8.3, 1.6 Hz, 1H), 7.71 (dd, *J*= 7.6, 1.1 Hz, 1H), 7.68–7.63 (m, 1H), 7.59 (dd, *J*= 8.3, 4.2 Hz, 1H), 7.56–7.47 (m, 2H), 7.44 (d, *J* = 2.2 Hz, 1H), 7.00 (d, *J*= 8.5 Hz, 1H), 3.74 (s, 3H), 3.69 (s, 3H). ^13 ^C NMR (100 MHz, DMSO-d_6_) *δ* 152.34, 149.30, 148.45, 138.67, 136.51, 133.76, 130.76, 128.05, 126.66, 122.77, 122.31, 120.84, 116.35, 110.91, 109.67, 55.72, 55.68. HRMS (m/z): Calcd. C_17_H_17_N_2_O_4_S, [M + H]^+^ m/z: 345.0909, found: 345.0914.

#### 4-Methoxy-N-(quinolin-8-yl)benzenesulphonamide (J2)

White solid, yield:87%, m.p.:1 6 1 ∼ 163 °C. ^1^H NMR (400 MHz, DMSO-d_6_) *δ* 9.79 (s, 1H), 8.87 (dd, *J*= 4.2, 1.6 Hz, 1H), 8.35 (dd, *J*= 8.3, 1.6 Hz, 1H), 7.98–7.80 (m, 2H), 7.77–7.41 (m, 4H), 7.09–6.91 (m, 2H), 3.74 (s, 3H). ^13 ^C NMR (100 MHz, DMSO-d_6_) *δ* 162.62, 149.30, 138.49, 136.51, 133.67, 130.83, 129.17, 128.03, 126.66, 122.67, 122.33, 115.94, 114.27, 55.57. HRMS (m/z): Calcd. C_16_H_15_N_2_O_3_S, [M + H]^+^ m/z: 315.0803, found: 315.0808.

#### 4-(Tert-butyl)-N-(quinolin-8-yl)benzenesulphonamide (J3)

White solid, yield:78%, m.p.:1 4 2 ∼ 144 °C. ^1^H NMR (400 MHz, DMSO-d_6_) *δ* 9.91 (s, 1H), 8.86 (dd, *J*= 4.2, 1.6 Hz, 1H), 8.35 (dd, *J*= 8.3, 1.6 Hz, 1H), 7.92 − 7.80 (m, 2H), 7.71 (dd, *J*= 7.6, 1.1 Hz, 1H), 7.65 (dd, *J*= 8.2, 1.0 Hz, 1H), 7.59 (dd, *J*= 8.3, 4.2 Hz, 1H), 7.55 − 7.46 (m, 3H), 1.20 (s, 9H). ^13 ^C NMR (100 MHz, DMSO-d_6_) *δ* 149.34, 139.41, 138.76, 136.48, 133.53, 133.07, 129.10, 128.06, 126.85, 126.62, 123.07, 122.30, 116.77. HRMS (m/z): Calcd. C_19_H_21_N_2_O_2_S, [M + H]^+^ m/z: 341.1324, found: 341.1329.

#### N-(quinolin-8-yl)benzenesulphonamide (J4)

White solid, yield:89%, m.p.:1 6 5 ∼ 167 °C. ^1^H NMR (400 MHz, DMSO-d_6_) *δ* 9.99 (s, 1H), 8.86 (dd, *J*= 4.2, 1.6 Hz, 1H), 8.35 (dd, *J*= 8.3, 1.5 Hz, 1H), 8.05–7.86 (m, 2H), 7.79–7.62 (m, 2H), 7.62–7.39 (m, 5H). ^13 ^C NMR (100 MHz, DMSO-d_6_) *δ* 156.15, 149.28, 138.54, 136.61, 136.49, 133.64, 128.07, 126.78, 126.68, 125.98, 122.75, 122.31, 116.02, 34.78, 30.62. HRMS (m/z): Calcd. C_15_H_13_N_2_O_2_S, [M + H]^+^ m/z: 285.0698, found: 285.0699.

#### 4-Methyl-N-(quinolin-8-yl)benzenesulphonamide (J5)

White solid, yield:84%, m.p.:1 4 8 ∼ 150 °C. ^1^H NMR (400 MHz, DMSO-d_6_) *δ* 9.88 (s, 1H), 8.87 (dd, *J*= 4.2, 1.5 Hz, 1H), 8.35 (dd, *J*= 8.3, 1.5 Hz, 1H), 7.81 (d, *J*= 8.3 Hz, 2H), 7.75–7.43 (m, 4H), 7.28 (d, *J*= 8.1 Hz, 2H), 2.27 (s, 3H). ^13 ^C NMR (100 MHz, DMSO-d_6_) *δ* 149.32, 143.56, 138.53, 136.52, 136.46, 133.58, 129.57, 128.04, 126.93, 126.65, 122.80, 122.33, 116.13, 20.86. HRMS (m/z): Calcd. C_16_H_15_N_2_O_2_S, [M + H]^+^ m/z: 299.0854, found: 299.0858.

#### 4-Bromo-N-(quinolin-8-yl)benzenesulphonamide (J6)

White solid, yield:92%, m.p.:1 9 2 ∼ 194 °C. ^1^H NMR (400 MHz, DMSO-d_6_) *δ* 10.19 (s, 1H), 8.85 (dd, *J*= 4.2, 1.6 Hz, 1H), 8.36 (dd, *J*= 8.3, 1.5 Hz, 1H), 7.83 (d, *J*= 8.6 Hz, 2H), 7.73–7.67 (m, 4H), 7.63–7.41 (m, 2H). ^13 ^C NMR (100 MHz, DMSO-d_6_) *δ* 149.41, 139.09, 138.87, 136.49, 133.35, 132.14, 128.90, 128.14, 126.90, 126.63, 123.51, 122.29, 117.68. HRMS (m/z): Calcd. C_15_H_12_BrN_2_O_2_S, [M + H]^+^ m/z: 362.9803, found: 362.9807.

#### N-(3,4,5-trimethoxyphenyl)quinoline-8-sulphonamide (J7)

White solid, yield:94%, m.p.:1 8 1 ∼ 183 °C. ^1^H NMR (400 MHz, DMSO-d_6_) *δ* 9.87 (s, 1H), 9.16 (dd, *J*= 4.2, 1.7 Hz, 1H), 8.52 (dd, *J*= 8.4, 1.7 Hz, 1H), 8.39 (dd, *J*= 7.3, 1.3 Hz, 1H), 8.27 (dd, *J*= 8.2, 1.3 Hz, 1H), 7.81–7.60 (m, 2H), 6.35 (s, 2H), 3.51 (s, 6H), 3.47 (s, 3H). ^13 ^C NMR (100 MHz, DMSO-d_6_) *δ* 152.59, 151.40, 142.72, 136.98, 135.12, 134.23, 133.92, 133.57, 132.25, 128.34, 125.63, 122.58, 98.02, 59.91, 55.56. HRMS (m/z): Calcd. C_18_H_19_N_2_O_5_S, [M + H]^+^ m/z: 375.1015, found: 375.1019.

#### N-(2-bromoethyl)-N-(3,4,5-trimethoxyphenyl)quinoline-8-sulphonamide (K1)

White solid, yield:72%, m.p.:1 5 8 ∼ 160 °C. ^1^H NMR (400 MHz, CDCl_3_) *δ* 9.11 (dd, *J*= 4.2, 1.8 Hz, 1H), 8.21 (dd, *J* = 8.6, 1.5 Hz, 2H), 7.94 (dd, *J*= 8.2, 1.3 Hz, 1H), 7.66–7.36 (m, 2H), 6.09 (s, 2H), 4.49 (t, *J*= 7.5 Hz, 2H), 3.67 (s, 3H), 3.48 (t, *J*= 7.5 Hz, 2H), 3.40 (s, 6H). ^13 ^C NMR (100 MHz, CDCl_3_) *δ* 152.07, 150.20, 143.11, 136.85, 135.75, 135.63, 133.29, 133.13, 132.51, 127.65, 124.69, 121.11, 105.47, 59.75, 54.85, 53.99, 29.17. HRMS (m/z): Calcd. C_20_H_22_BrN_2_O_5_S, [M + H]^+^ m/z: 481.0433, found: 481.0439.

#### N-(3-bromopropyl)-N-(3,4,5-trimethoxyphenyl)quinoline-8-sulphonamide (K2)

White solid, yield:81%, m.p.:1 2 9 ∼ 130 °C. ^1^H NMR (400 MHz, CDCl_3_) *δ* 9.09 (dd, *J*= 4.2, 1.7 Hz, 1H), 8.20 (td, *J*= 8.6, 1.5 Hz, 2H), 7.93 (dd, *J*= 8.2, 1.2 Hz, 1H), 7.62–7.32 (m, 2H), 6.12 (s, 2H), 4.22 (t, *J*= 6.8 Hz, 2H), 3.67 (s, 3H), 3.47 (t, *J*= 6.8 Hz, 2H), 3.42 (d, *J*= 8.3 Hz, 6H), 2.09 (p, *J*= 6.8 Hz, 2H). ^13 ^C NMR (100 MHz, CDCl_3_) *δ* 152.00, 150.12, 143.22, 136.55, 135.78, 135.56, 133.61, 133.14, 132.46, 127.67, 124.63, 121.03, 105.19, 59.77, 54.89, 51.02, 31.75, 29.45. HRMS (m/z): Calcd. C_21_H_24_BrN_2_O_5_S, [M + H]^+^ m/z: 495.0589, found: 495.0593.

#### N-(4-bromobutyl)-N-(3,4,5-trimethoxyphenyl)quinoline-8-sulphonamide (K3)

White solid, yield:96%, m.p.:1 0 3 ∼ 104 °C. ^1^H NMR (400 MHz, CDCl_3_) *δ* 9.08 (dd, *J*= 4.2, 1.8 Hz, 1H), 8.19 (ddd, *J*= 6.0, 3.9, 1.6 Hz, 2H), 7.92 (dd, *J*= 8.2, 1.3 Hz, 1H), 7.60–7.31 (m, 2H), 6.08 (s, 2H), 4.12 (t, *J*= 6.8 Hz, 2H), 3.67 (s, 3H), 3.53–3.33 (m, 8H), 2.01 (dt, *J*= 14.4, 6.6 Hz, 2H), 1.64 (dt, *J*= 14.1, 6.9 Hz, 2H). ^13 ^C NMR (100 MHz, CDCl_3_) *δ* 151.95, 150.08, 143.25, 136.54, 136.01, 135.52, 133.50, 133.06, 132.31, 127.64, 124.63, 120.98, 105.48, 59.76, 54.87, 51.59, 32.67, 28.66, 26.64. HRMS (m/z): Calcd. C_22_H_26_BrN_2_O_5_S, [M + H]^+^ m/z: 509.0746, found: 509.0748.

#### Tert-butyl-4-(((2-(N-(3,4,5-trimethoxyphenyl)quinoline-8-sulfonamido)ethyl)thio)carbonothioyl)piperazine-1-carboxylate (L1)

White solid, yield:62%, m.p.:1 2 1 ∼ 123 °C. ^1^H NMR (400 MHz, CDCl_3_) *δ* 9.07 (dd, *J*= 4.2, 1.8 Hz, 1H), 8.21 (ddd, *J*= 10.1, 7.9, 1.5 Hz, 2H), 7.93 (dd, *J*= 8.2, 1.3 Hz, 1H), 7.58–7.34 (m, 2H), 6.17 (s, 2H), 4.41 (t, *J*= 7.0 Hz, 2H), 4.34–4.06 (m, 2H), 4.04–3.76 (m, 2H), 3.67 (s, 3H), 3.52 (t, *J*= 7.0 Hz, 2H), 3.47 (dd, *J*= 10.0, 5.0 Hz, 4H), 3.44 (s, 6H), 1.41 (s, 9H). ^13 ^C NMR (100 MHz, CDCl_3_) *δ* 195.89, 153.45, 151.90, 150.18, 143.16, 136.51, 135.93, 135.52, 133.33, 133.03, 132.43, 127.64, 124.62, 121.01, 105.28, 79.58, 59.77, 54.91, 50.77, 35.22, 27.35. HRMS (m/z): Calcd. C_30_H_39_N_4_O_7_S_3_, [M + H]^+^ m/z: 663.1981, found: 663.1987.

#### Tert-butyl-4-(((3-(N-(3,4,5-trimethoxyphenyl)quinoline-8-sulfonamido)propyl)thio)carbonothioyl)piperazine-1-carboxylate (L2)

White solid, yield:91%, m.p.:1 3 0 ∼ 132 °C. ^1^H NMR (400 MHz, CDCl_3_) *δ* 9.09 (dd, *J*= 4.2, 1.8 Hz, 1H), 8.35–8.06 (m, 2H), 7.92 (dd, *J*= 8.2, 1.3 Hz, 1H), 7.63–7.35 (m, 2H), 6.13 (s, 2H), 4.20 (t, *J*= 6.7 Hz, 4H), 3.91 (s, 2H), 3.67 (s, 3H), 3.51–3.44 (m, 4H), 3.45–3.36 (m, 8H), 2.00–1.85 (m, 2H), 1.40 (s, 9H). ^13 ^C NMR (100 MHz, CDCl_3_) *δ* 196.61, 153.47, 151.94, 150.17, 143.24, 136.50, 135.97, 135.51, 133.57, 133.07, 132.34, 127.64, 124.61, 121.01, 105.45, 79.56, 59.75, 54.90, 51.54, 33.18, 27.76, 27.35. HRMS (m/z): Calcd. C_31_H_41_N_4_O_7_S_3_, [M + H]^+^ m/z: 677.2137, found: 677.2139.

#### 2-(N-(3,4,5-trimethoxyphenyl)quinoline-8-sulfonamido)ethyl-4–(2-hydroxyethyl)piperazine-1-carbodithioate (L3)

White solid, yield:89%, m.p.:1 2 2 ∼ 124 °C. ^1^H NMR (400 MHz, DMSO-d_6_) *δ* 9.16 (dd, *J*= 4.1, 1.5 Hz, 1H), 8.57 (dd, *J*= 8.4, 1.4 Hz, 1H), 8.29 (d, *J*= 7.5 Hz, 1H), 8.21 (d, *J* = 7.3 Hz, 1H), 7.75 (dd, *J*= 8.3, 4.2 Hz, 1H), 7.65 (t, *J*= 7.8 Hz, 1H), 6.24 (s, 2H), 4.48 (t, *J* = 5.3 Hz, 1H), 4.35 (t, *J*= 6.8 Hz, 2H), 4.19 (s, 2H), 3.86 (s, 2H), 3.55 (d, *J*= 6.5 Hz, 3H), 3.52 (dd, *J*= 11.5, 5.9 Hz, 2H), 3.42 (s, 6H), 3.39 (d, *J*= 6.8 Hz, 2H), 2.50–2.46 (m, 4H), 2.43 (t, *J*= 6.1 Hz, 2H). ^13 ^C NMR (100 MHz, DMSO-d_6_) *δ* 194.23, 152.42, 151.54, 143.26, 137.00, 136.75, 136.02, 134.24, 133.97, 133.38, 128.49, 125.67, 122.56, 106.00, 59.95, 59.45, 58.46, 55.55, 52.47, 50.94, 35.54. HRMS (m/z): Calcd. C_27_H_35_N_4_O_6_S_3_, [M + H]^+^ m/z: 607.1719, found: 607.1723.

#### 3-(N-(3,4,5-trimethoxyphenyl)quinoline-8-sulfonamido)propyl-4–(2-hydroxyethyl)piperazine-1-carbodithioate (L4)

White solid, yield:85%, m.p.:1 0 6 ∼ 107 °C. ^1^H NMR (400 MHz, CDCl_3_) *δ* 9.10 (d, *J*= 2.1 Hz, 1H), 8.20 (dd, *J*= 6.8, 3.9 Hz, 2H), 7.93 (d, *J*= 8.1 Hz, 1H), 7.65–7.34 (m, 2H), 6.12 (s, 2H), 4.33 (s, 2H), 4.20 (t, *J*= 6.6 Hz, 2H), 3.96 (s, 2H), 3.67 (s, 3H), 3.66–3.57 (m, 2H), 3.42 (s, 8H), 2.73–2.49 (m, 6H), 1.93 (dd, *J* = 13.7, 6.8 Hz, 2H). ^13 ^C NMR (100 MHz, CDCl_3_) *δ* 196.25, 151.89, 150.19, 143.20, 136.34, 135.86, 135.53, 133.54, 133.08, 132.36, 127.61, 124.62, 121.02, 105.31, 59.76, 58.16, 56.69, 54.85, 51.52, 51.28, 33.21, 27.73. HRMS (m/z): Calcd. C_28_H_37_N_4_O_6_S_3_, [M + H]^+^ m/z: 621.1875, found: 621.1879.

#### 4-(N-(3,4,5-trimethoxyphenyl)quinoline-8-sulfonamido)butyl-4-ethylpiperazine-1-carbodithioate (L5)

White solid, yield:85%, m.p.:1 1 0 ∼ 112 °C. ^1^H NMR (400 MHz, CDCl_3_) *δ* 9.08 (dd, *J*= 4.2, 1.8 Hz, 1H), 8.28–8.09 (m, 2H), 7.92 (dd, *J*= 8.2, 1.3 Hz, 1H), 7.61–7.33 (m, 2H), 6.09 (s, 2H), 4.28 (s, 2H), 4.10 (t, *J*= 7.0 Hz, 2H), 3.90 (s, 2H), 3.68 (s, 3H), 3.43 (s, 6H), 3.26 (t, *J*= 7.4 Hz, 2H), 2.60–2.42 (m, 4H), 2.39 (q, *J*= 7.2 Hz, 2H), 1.79 (dd, *J*= 14.9, 8.0 Hz, 2H), 1.67–1.59 (m, 2H), 1.04 (t, *J*= 7.2 Hz, 3H). ^13 ^C NMR (100 MHz, CDCl_3_) *δ* 195.96, 151.90, 150.12, 143.25, 136.45, 136.14, 135.46, 133.62, 132.95, 132.25, 127.64, 124.58, 120.97, 105.52, 59.75, 54.88, 52.07, 51.15, 50.89, 35.65, 27.66, 25.04, 10.96. HRMS (m/z): Calcd. C_29_H_39_N_4_O_5_S_3_, [M + H]^+^ m/z: 619.2083, found: 619.2087.

#### Tert-butyl-4-(((4-(N-(3,4,5-trimethoxyphenyl)quinoline-8-sulfonamido)butyl)thio)carbonothioyl)piperazine-1-carboxylate (L6)

White solid, yield:74%, m.p.:9 8 ∼ 100 °C. ^1^H NMR (400 MHz, CDCl_3_) *δ* 9.08 (dd, *J*= 4.2, 1.7 Hz, 1H), 8.31–8.13 (m, 2H), 7.92 (dd, *J*= 8.2, 1.2 Hz, 1H), 7.59–7.38 (m, 2H), 6.09 (s, 2H), 4.10 (t, *J*= 7.0 Hz, 6H), 3.67 (s, 3H), 3.54–3.46 (m, 4H), 3.42 (s, 6H), 3.27 (t, *J*= 7.4 Hz, 2H), 1.86–1.75 (m, 2H), 1.66–1.58 (m, 2H), 1.41 (s, 9H). ^13 ^C NMR (100 MHz, CDCl_3_) *δ* 157.80, 153.47, 151.90, 150.11, 143.23, 136.46, 136.09, 135.47, 133.59, 132.97, 132.27, 127.65, 124.61, 120.97, 105.49, 79.55, 59.75, 54.87, 52.07, 35.65, 27.62, 27.35, 24.95. HRMS (m/z): Calcd. C_32_H_43_N_4_O_7_S_3_, [M + H]^+^ m/z: 691.2294, found: 691.2298.

#### 4-(N-(3,4,5-trimethoxyphenyl)quinoline-8-sulfonamido)butyl-4-methylpiperazine-1-carbodithioate (L7)

White solid, yield:69%, m.p.:1 3 5 ∼ 137 °C. ^1^H NMR (400 MHz, DMSO) *δ* 9.17 (dd, *J*= 4.2, 1.7 Hz, 1H), 8.56 (dd, *J*= 8.4, 1.6 Hz, 1H), 8.23 (ddd, *J*= 42.3, 7.8, 1.2 Hz, 2H), 7.78–7.59 (m, 2H), 6.17 (s, 2H), 4.23 (s, 2H), 4.08 (t, *J*= 6.7 Hz, 2H), 3.88 (s, 2H), 3.56 (s, 3H), 3.41 (s, 6H), 3.24 (t, *J*= 7.3 Hz, 2H), 2.44–2.29 (m, 4H), 2.21 (s, 3H), 1.72 (dd, *J*= 14.7, 7.7 Hz, 2H), 1.52 (dd, *J*= 14.5, 6.9 Hz, 2H). ^13 ^C NMR (100 MHz, DMSO-d_6_) *δ* 195.24, 152.43, 151.46, 143.31, 136.92, 136.71, 136.27, 134.27, 134.04, 133.23, 128.48, 125.62, 122.49, 106.15, 59.93, 55.52, 53.94, 52.16, 45.05, 35.70, 27.83, 25.74. HRMS (m/z): Calcd. C_28_H_37_N_4_O_5_S_3_, [M + H]^+^ m/z: 605.1926, found: 605.1929.

#### 4-(N-(3,4,5-trimethoxyphenyl)quinoline-8-sulfonamido)butyl-4–(2-hydroxyethyl)piperazine-1-carbodithioate (L8)

White solid, yield:73%, m.p.:9 4 ∼ 96 °C. ^1^H NMR (400 MHz, CDCl_3_) *δ* 9.08 (dd, *J*= 4.1, 1.6 Hz, 1H), 8.19 (dd, *J*= 10.7, 3.9 Hz, 2H), 7.92 (d, *J*= 7.3 Hz, 1H), 7.53–7.40 (m, 2H), 6.09 (s, 2H), 4.28 (s, 2H), 4.10 (t, *J*= 7.0 Hz, 2H), 3.88 (d, *J*= 20.8 Hz, 2H), 3.67 (s, 3H), 3.64–3.57 (m, 2H), 3.42 (s, 6H), 3.26 (t, *J*= 7.4 Hz, 2H), 2.53 (dd, *J*= 6.2, 3.8 Hz, 6H), 1.80 (dd, *J*= 14.8, 7.7 Hz, 2H), 1.65–1.55 (m, 2H). ^13 ^C NMR (100 MHz, CDCl_3_) *δ* 196.27, 151.91, 150.12, 143.25, 136.45, 136.13, 135.47, 133.61, 132.97, 132.25, 127.64, 124.60, 120.97, 105.52, 59.76, 58.04, 56.96, 54.88, 52.08, 51.32, 35.69, 27.63, 25.01. HRMS (m/z): Calcd. C_29_H_39_N_4_O_6_S_3_, [M + H]^+^ m/z: 635.2032, found: 635.2037.

### Inhibitory evaluation of compounds J1∼J7, K1∼K3, and L1∼L8 against LSD1 and MAO-A/B

LSD1 inhibitor screening assay kit was purchased to evaluate for the enzyme inhibition activity of all synthetic compounds (Cayman Chemical, Fort Annapolis, MI). The MAO-A/B biochemical kit was purchased to explore the inhibitory activity against MAO-A and MAO-B (Promega, Madison, WI). First, compounds **J1∼J7**, **K1∼K3,** and **L1∼L8** were diluted with dimethyl sulphoxide to required concentrations (Innochem, Beijing, China). In a 48-well black plate, a buffer solution of compounds and reagent was prepared and incubated at 37 °C. Then, horseradish peroxidase solution and fluorometric substrate solution were added and incubated at 37 °C. The signal of each well was read by a GloMax Explorer (Beijing Yuanpinghao Biotechnology Co., Ltd, Beijing, China).

### Cell culture and cell viability assay

Cell lines (Bel7402, H1299, HCT-15, SGC-7901, HTB3, Jurkat, LX-2, HPAEpiC, HIEC, HTB5, HT1376, and HTB1) were obtained from Shanghai Yuanye Biotechnology Co., Ltd (Shanghai, China). Cell lines were cultured in 89% Roswell Park Memorial Institute 1640 medium supplemented with 10% foetal bovine serum and 1% antibiotic at 37 °C in a humidified CO_2_ incubator (Shanghai Yuanye Biotechnology Co., Ltd, Shanghai, China). HTB3&shControl, HTB3&shLSD1, HT1376&shControl, and HT1376&shLSD1 cells were supported by the 4th affiliated Hospital of China Medical University. HTB3&shControl, HTB3&shLSD1, HT1376&shControl, and HT1376&shLSD1 cells were cultured in a Roswell Park Memorial Institute 1640 medium with geneticin (Innochem, Beijing, China). Cells were cultured at a 96-well plate for 24 h (Cayman Chemical, Fort Annapolis, MI) and different compounds were added. Thiazolyl blue tetrazolium bromide (Innochem, Beijing, China) was added and cultured for 4 h at 37 °C in a humidified CO_2_ incubator. The solution was removed and dimethyl sulphoxide was added. Thirty minutes later, absorption values were read by a Fully Automated Microplate ELISA Analyser (DeTie, NanJing, China).

### LSD1 dilution assay

LSD1 recombinant (Cayman Chemical, Fort Annapolis, MI) was incubated with the targeted compound or dimethyl sulphoxide for 60 min. Then, samples were diluted 80 times using a HRP-assay solution containing substrate and coupling reagents. Finally, LSD1 inhibitory activity before and after dilution was examined.

### Western blotting

Cells were seeded in four-well plates (Shanghai Yuanye Biotechnology Co., Ltd, Shanghai, China) and exposed to different compounds at different concentrations. Cells were harvested to obtain protein solution by the RIPA Lysis Buffer (DeTie, NanJing, China). The proteins were separated on sodium dodecyl sulphate–polyacrylamide gel electrophoresis (SDS-PAGE) gels and then transferred to poly(vinylidene fluoride (PVDF) membranes (Shanghai Yuanye Biotechnology Co., Ltd, Shanghai, China). It incubated with the primary antibodies and corresponding HRP conjugated secondary antibodies (Servicebio, Wuhan, China). The electrochemiluminescence kit (Servicebio, Wuhan, China) was used to visualise the bands.

### Molecular docking analysis

The protein structure of LSD1 was obtained from Protein Data Bank (PDB code: 2v1d). Sulphonamide derivatives **L6∼L8** were transferred as PDB files by ChemBio 3 D Ultra 14.0 (CambridgeSoft, Cambridge, MA). Water was removed from LSD1 protein by Pymol. Autodock (The Scripps Research Institute, San Diego, CA) was used to perform antogrid and autodock. The hydrogen-bond interaction between sulphonamide derivatives **L6∼L8** and amino acid residues of LSD1 was analyzed^26^^,^^27^.

### Statistical methods

All data of biological experiments were expressed as the mean ± SD. Significant differences between different groups were analysed by GraphPad Prism version 6 (GraphPad Software, La Jolla, CA). Results were considered statistically significant at **p< 0.01 verse control, ***p< 0.001 verse control and ^****^p< 0.0001 verse control.

## Results and discussion

### Synthesis of sulphonamide derivatives

**Scheme 1.**
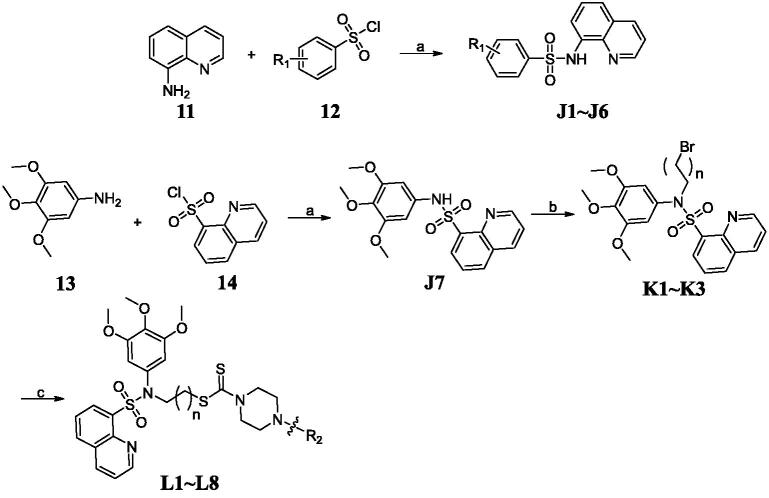
Synthesis of the sulphonamide derivatives. Reagents and conditions: (a) triethylamine, acetone, 0 °C; (b) 1,2-dibromoethane or 1,3-dibromopropane or 1,4-dibromobutane, dibromobutane, KOH, 60 °C; (c) CS_2_, piperazine derivatives, and trisodium phosphate dodecahydrate.

Although LSD1 inhibition has been a promising strategy to treat cancer, only few LSD1 inhibitors are currently in clinical trials. Discovery of novel and effective scaffolds to develop LSD1 inhibitors has an important clinical significance. Our aim was to synthesise and identify novel LSD1 inhibitors. Sulphonamide derivatives were synthesised as shown in Scheme 1. Commercially available quinolin-8-amine was treated with different benzenesulfonyl chloride to provide **J1**∼**J6** and 3,4,5-trimethoxyaniline was treated with quinoline-8-sulphonyl chloride to afford **J7**. Compounds **K1**∼**K3** were obtained by the alkylation of sulphonamide **J7** with 1,2-dibromoethane or 1,3-dibromopropane or 1,4-dibromobutane. Sulphonamide intermediates **K1**∼**K3** were treated with carbon disulphide and piperazine derivatives in the presence of trisodium phosphate dodecahydrate to obtain sulphonamide-quinoline-dithiocarbamate hybrids **L1**∼**L8**.

### LSD1 inhibition and preliminary structure activity relationship

All sulphonamide derivatives in this work were examined *in vitro* for their inhibitory activity against LSD1, MAO-A, and MAO-B. 2-PCPA (tranylcypromine) and ORY-1001 as LSD1 inhibitors were chosen as reference agents^28^. The inhibitory results of sulphonamides **J1**∼**J7** were summarised in Table 1. Sulphonamide derivatives **J1**∼**J7** had very weak inhibition effects on MAO-A and MAO-B with IC_50_ values of >120 μM. However, all sulphonamide derivatives **J1**∼**J7** displayed the moderate activity against LSD1 with IC_50_ values ranging from 11.57 to 82.46 μM. Substituent groups on phenyl ring could affect the inhibitory activity against LSD1. Among all these sulphonamides, *N*-(3,4,5-trimethoxyphenyl)quinoline-8-sulphonamide **J7** exhibited the best inhibitory activity, indicating that sulphonamide-quinoline might be a potential scaffold to design LSD1 inhibitors.

**Table 1.** Inhibitory activity of sulphonamide derivatives **J1**∼**J7** against LSD1, MAO-A, and MAO-B. 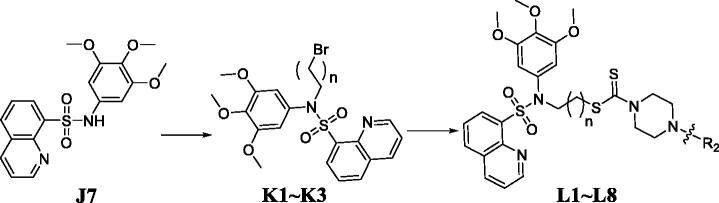

Compound	R_1_	IC_50_ (μM)		
		LSD1	MAO-A	MAO-B
**J1**	3,4-diOCH_3_	33.78 ± 2.04	>120	>120
**J2**	4-OCH_3_	45.62 ± 1.12	>120	>120
**J3**	4-C(CH_3_)_3_	61.90 ± 2.46	>120	>120
**J4**	H	82.46 ± 3.38	>120	>120
**J5**	4-CH_3_	74.52 ± 2.67	>120	>120
**J6**	4-Br	61.82 ± 5.64	>120	>120
**J7**	–	11.57 ± 1.92	>120	>120
**2-PCPA**	–	25.63 ± 2.76	14.57 ± 1.29	6.47 ± 0.62
**ORY-1001**	–	0.03 ± 0.01	>120	>120

Because **J7** showed the best inhibitory activity against LSD1, more sulphonamide analogues were designed and synthesised based on compound **J7**. The *in vitro* inhibitory activity results of sulphonamides **K1**∼**K3** and **L1**∼**L8** were listed in Table 2. With the exception of sulphonamide–quinoline–dithiocarbamate hybrids **L1**∼**L8**, all these sulphonamide analogues bearing a dithiocarbamate fragment exhibit potently inhibitory activity with IC_50_ values ranging from 0.06 to 4.92 μM. In comparison with activity results of sulphonamide intermediates **K1**∼**K3** without the dithiocarbamate unit, the inhibitory activity of sulphonamide–quinoline–dithiocarbamate hybrids **L1**∼**L8** against LSD1 improved obviously, indicating that the dithiocarbamate scaffold play a synergistic role on LSD1 activity. Especially, compound **L8** showed the best activity against LSD1, with an IC_50_ of 0.06 μM, which is 427 times higher than that of 2-PCPA. During the preliminary structure activity relationship studies, we found that the substituent on the piperazine ring was significant for the LSD1 inhibitory activity showing over 80-fold activity loss, when the hydroxyethyl group was replaced with the ethyl group (compound **L8** *versus* **L5**). When the substituent on the piperazine ring was tert-butoxycarbonyl group and methyl group, compound **L6** and compound **L7** displayed the potent LSD1 inhibitory activity with IC_50_ values of 0.23 and 0.73 μM, respectively. In addition, the length of carbon linker between the sulphonamide and the dithiocarbamate exhibited a significant role in their activities. With the reduction of the carbon tether length, a decrease of LSD1 inhibitory activity was observed (compound **L8** *versus* **L3**, or compound **L6** *versus* **L1**). Based on the enzyme inhibitory results, all sulphonamide derivatives **L1**∼**L8** potently inhibited LSD1 with IC_50_ values ranging from 4.92 to 0.06 µM. Importantly, they had no significant effects on MAO-A and MAO-B with IC_50_ values of >120 µM, indicating that these novel sulphonamide derivatives were selective LSD1 inhibitors. For the preliminary structure activity relationships, dithiocarbamate unit and substituent groups on piperazine of sulphonamide derivatives **L1**∼**L8** played significant roles on LSD1 inhibitory activity.

**Table 2.** Inhibitory activity of sulphonamide derivatives **K1**∼**K3** and **L1**∼**L8** against LSD1, MAO-A, and MAO-B. 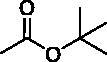

Compound	n	R_2_	IC_50_ (μM)		
			LSD1	MAO-A	MAO-B
**J7**	–	–	11.57 ± 1.92	>120	>120
**K1**	1	–	14.79 ± 2.04	>120	>120
**K2**	2	–	9.63 ± 1.26	>120	>120
**K3**	3	–	6.32 ± 0.77	>120	>120
**L1**	1	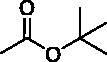	2.73 ± 0.29	>120	>120
**L2**	2	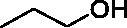	1.39 ± 0.14	>120	>120
**L3**	1	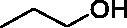	1.85 ± 0.07	>120	>120
**L4**	2	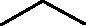	0.67 ± 0.10	>120	>120
**L5**	3	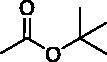	4.92 ± 0.13	>120	>120
**L6**	3	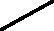	0.23 ± 0.05	>120	>120
**L7**	3	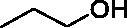	0.73 ± 0.08	>120	>120
**L8**	3		0.06 ± 0.01	>120	>120
**2-PCPA**	–	–	25.63 ± 2.76	14.57 ± 1.29	6.47 ± 0.62
**ORY-1001**	–	–	0.03 ± 0.01	>120	>120

### Sulphonamide derivative L8 exhibited good selectivity between cancer cells and normal cells

Based on the LSD1 inhibitory activity results of all sulphonamide derivatives, the most potent LSD1 inhibitor **L8** was prioritised to perform further experiments for evaluating its antiproliferative effects against cancer cell lines and normal cell lines. Bel7402 (human liver cell line), H1299 (human lung cell line), HCT-15 (human colon cell line), SGC-7901 (human gastric cell line), HTB3 (human bladder cell line), Jurkat (human leukaemia cell line), LX-2 (normal human hepatic stellate cell line), HPAEpiC (normal human alveolar epithelial cell line), and HIEC (normal human intestinal epithelial cell line) were selected to do MTT assay with the treatment of compound **L8** for 48 h. The IC_50_ values of compound **L8** were 0.96, 0.31, 2.17, 2.67, 0.18, and 0.29 μM against Bel7402, H1299, HCT-15, SGC-7901, HTB3, and Jurkat cells, respectively. However, compound **L8** showed weak antiproliferative activity against normal cell lines (LX-2, HPAEpiC, and HIEC) with IC_50_ values of >16 μM. Thus, sulphonamide derivative **L8** exhibited good selectivity between cancer cells and normal cells.

### Sulphonamide derivative L8 potently inhibited cell proliferation against bladder cancer in a concentration-dependent and time-dependent manner

Among all six cancer cell lines, sulphonamide derivative **L8** displayed the best activity results against bladder cancer HTB3 cells. So, bladder cancer was chosen to investigate the mechanisms of compound **L8**. According to results, sulphonamide derivative **L8** potently inhibited cell proliferation against different bladder cancer cell lines. When these four bladder cancer cell lines were treated with compound **L8**, IC_50_ values for 48 h were 1.87, 0.18, 0.09, and 0.93 μM against HTB5, HTB3, HT1376, and HTB1 cells, respectively. In addition, cell viability of bladder cancer decreased obviously in a concentration-dependent and time-dependent manner.

### Sulphonamide derivative L8 selectively and reversibly inhibited LSD1 in enzyme-based assays

**Figure 3.**
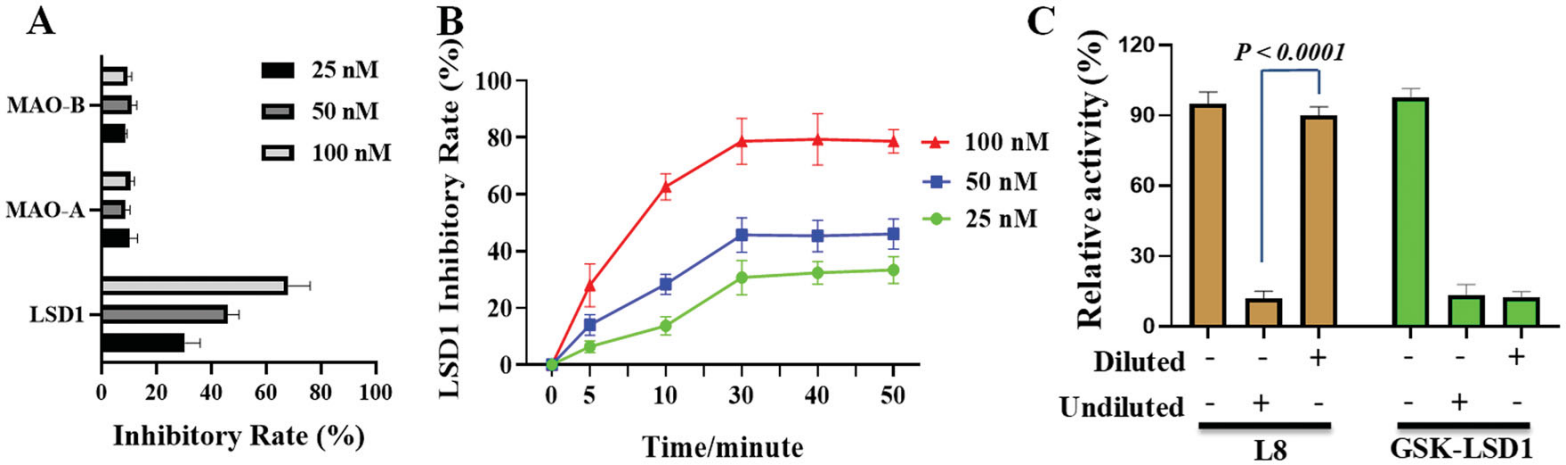
Mechanism studies of sulphonamide derivative **L8** on LSD1 inhibitory activity at the enzyme level. (A) Enzyme selectivity of sulphonamide derivative **L8**. (B) Time dependent curve of LSD1 inhibition by compound **L8**. (C) Reversible study of compound **L8** against LSD1. GSK-LSD1 was a famous LSD1 inhibitor^29^.

From the enzyme inhibitory results in Figure 3(A**)**, sulphonamide derivative L8 weakly inhibited MAO-A and MAO-B with the inhibitory rates of only 10.33% and 9.33% at 100 nM, while it showed about 30.33%, 46.00%, and 68.00% of inhibition against LSD1 at 25, 50, and 100 nM, suggesting that compound **L8** selectively inhibited LSD1 in a concentration-dependent. As shown in Figure 3(B), sulphonamide derivative **L8** potently inhibited LSD1 in a time-dependent manner. In the dilution assay, the low concentration of sulphonamide derivative **L8** by the dilution could result in the recovery of LSD1 inhibitory activity. Results in Figure 3(C) showed that sulphonamide derivative **L8** was a reversible LSD1 inhibitor.

### Sulphonamide derivative L8 potently inhibited LSD1 in cell-based assays

**Figure 4.**
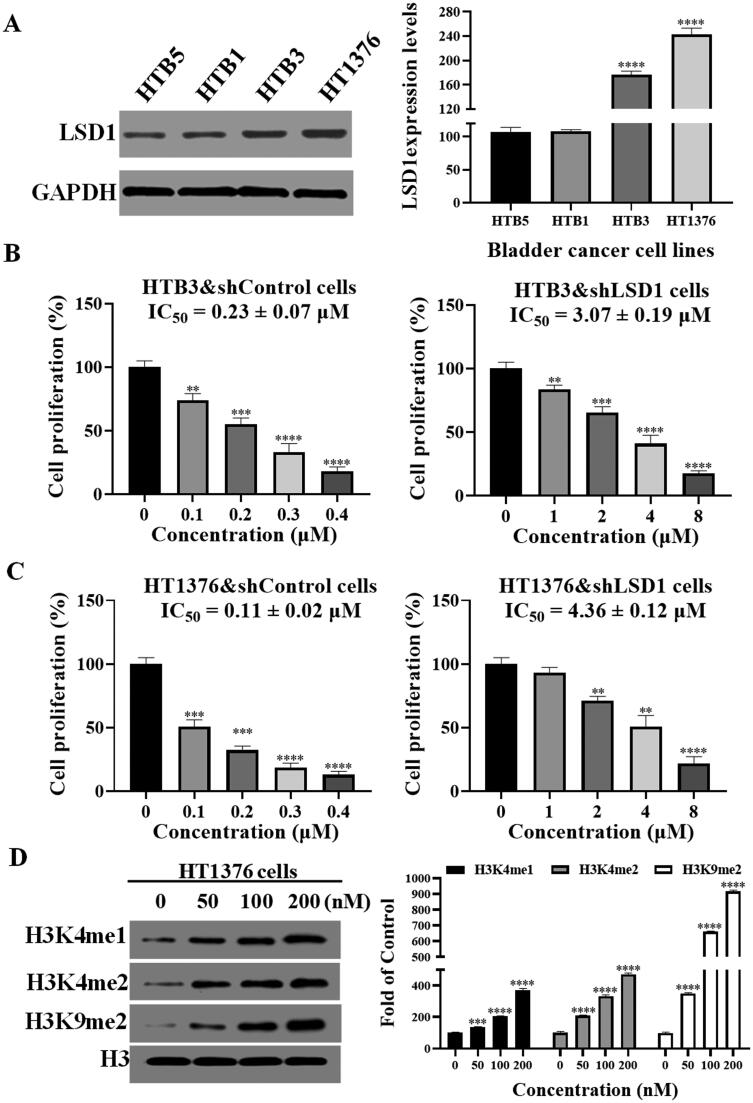
Mechanism studies of sulphonamide derivative **L8** on LSD1 inhibitory activity at the cellular level. (A) LSD1expression levels against bladder cancer cell lines. (B) Cell proliferation (%) of HTB3&shControl cells and HTB3&shLSD1 cells. (C) Cell proliferation (%) of HT1376&shControl cells and HT1376&shLSD1 cells. (D) The expression levels of H3K4me1, H3K4me2, and H3K9me2 against HT1376 cells with the treatment of **L8**. **P < 0.01 verse control, ***p< 0.001 verse control and ^****^p< 0.0001 verse control.

Before investigating the LSD1 inhibitory mechanisms at the cellular level, we first examined the LSD1 expression levels in four different bladder cancer cell lines (HTB5, HTB1, HTB3, and HT1376). From the results in Figure 4(A), HT1376 cells possessed the highest LSD1 expression levels, followed by HTB3, HTB1, and HTB5. In this work, we used the LSD1 knock-down cells and control cells to investigate the *in vitro* antiproliferative activity of LSD1 inhibitor **L8** (Figure 4(B,C)). Compound **L8** inhibited HTB3&shLSD1 cells and HT1376&shLSD1 cells with the IC_50_ values of 3.07 and 4.36 μM. However, it significantly inhibited cell proliferation against HTB3&shControl cells and HT1376&shControl cells with the IC_50_ values of 0.23 and 0.11 μM, respectively. The great activity discrepancy between LSD1 knock-down cells and control cells showed that the antiproliferative effects of sulphonamide derivative **L8** against bladder cancer was dependent on its LSD1 inhibition. Meanwhile, several substrates of LSD1, including H3K4me1, H3K4me2, and H3K9me2 were also investigated to their expression levels with the compound treatment. As shown in Figure 4(D), sulphonamide derivative **L8** increased the expression levels of H3K4me1, H3K4me2, and H3K9me2 against HT1376 cells. All these results showed that sulphonamide derivative **L8** could inhibit LSD1 in the cellular level.

### Molecular docking analysis of sulphonamide derivative L8 targeting LSD1

**Figure 5.**
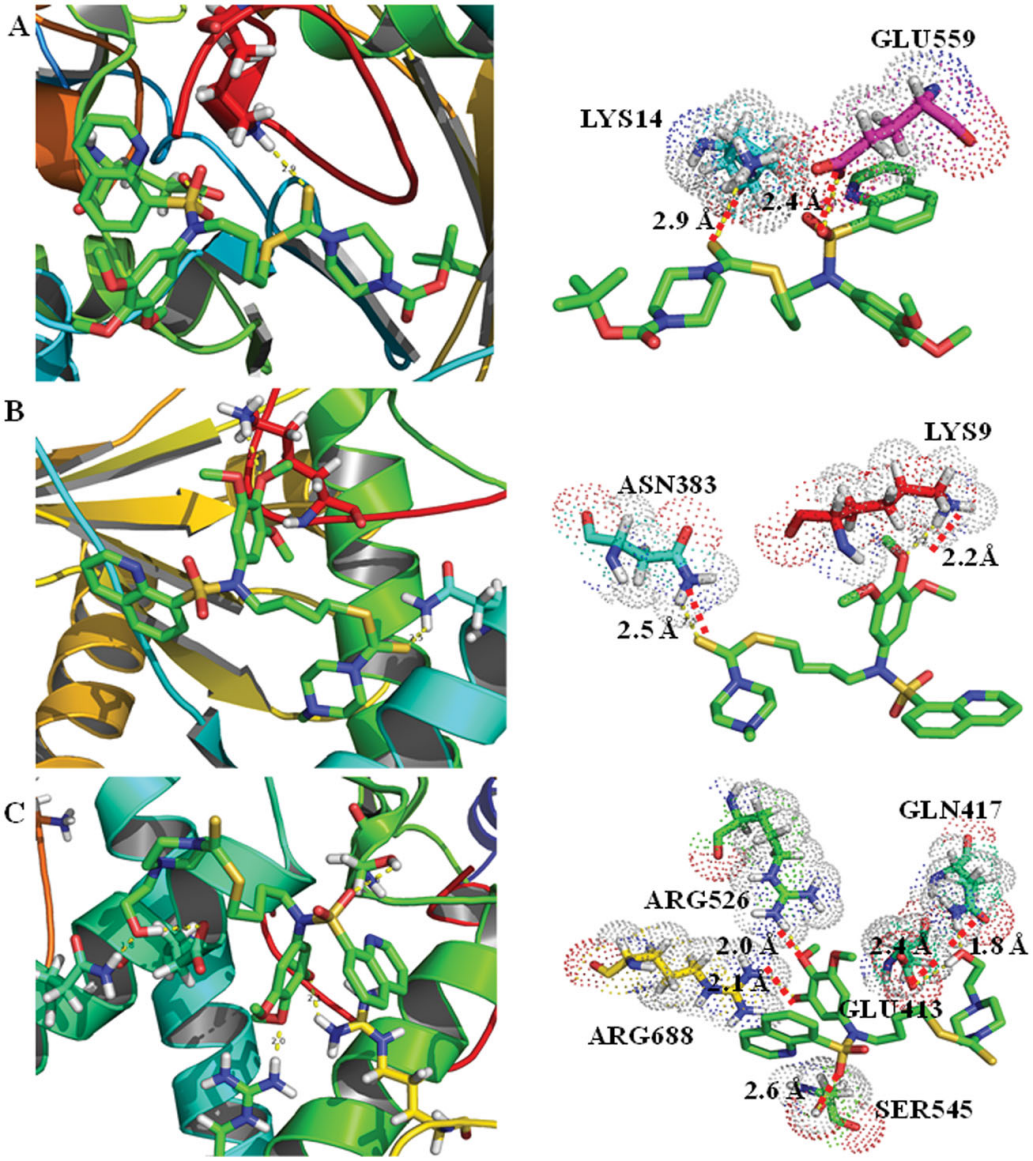
Molecular docking results of sulphonamides **L6**∼**L8** in binding with LSD1. (A) Binding interactions between sulphonamide **L6** and LSD1. (B) Binding interactions between sulphonamide **L7** and LSD1. (C) Binding interactions between sulphonamide **L8** and LSD1.

A molecular docking was carried out to predict the binding interaction between sulphonamide derivatives and LSD1. The protein structure of LSD1 was extracted from Protein Data Bank (PDB code: 2V1D, resolution: 3.1 Å) and was prepared by adding hydrogen atoms and removing flavin adenine dinucleotide. The molecular docking results in Figure 5 indicated that sulphonamide derivatives **L6**∼**L8** showed different binding modes through hydrogen bonds in the active pocket of LSD1. The dithiocarbamate group and amide group of sulphonamide derivative **L6** formed two hydrogen bonds with LYS14 and GLU559, and the distance was 2.9 and 2.4 Å, respectively. In addition, compound **L7** also had hydrogen interactions with LYS9 and ASN383 and the distance was 2.2 and 2.5 Å, respectively. Compared with compound **L6** and **L7**, sulphonamide derivative **L8** formed more hydrogen bonds with residues of LSD1. Compound **L8** makes five hydrogen bonds with amino acid residues GLN417, GLU413, SER545, ARG688, and ARG526. The docking studies of sulphonamide derivatives were in accordance with the results of LSD1 inhibitory activity.

## Conclusion

In conclusion, we have designed and synthesised a new class of sulphonamide–quinoline–dithiocarbamate hybrids as LSD1 inhibitors. Novel sulphonamide derivative **L8** selectively and reversibly inhibits LSD1 in a concentration-dependent and time-dependent manner. Importantly, sulphonamide derivative **L8** as a potent antiproliferative agent suppresses the proliferation against bladder cancer cells. To the best of our knowledge, there have been no literature reports regarding sulphonamide–quinoline–dithiocarbamate hybrids as LSD1 inhibitors against bladder cancer cells so far. All these findings provide an effective molecular skeleton for the discovery of LSD1 inhibitors, and sulphonamide based LSD1 inhibitors might be potentially anticancer drugs to treat bladder cancer.

## Supplementary Material

Supplemental Material
