# Supplementary material for: Discovery of novel sulphonamide hybrids that inhibit LSD1 against bladder cancer cells
Source: J Enzyme Inhib Med Chem. 2022 Mar 30;37(1):866–75. doi: 10.1080/14756366.2021.2014830 (PMC8973347; doi:10.1080/14756366.2021.2014830)
Supplement: Supplemental Material [file IENZ_A_2014830_SM6294.pdf]

Discovery of novel sulfonamide hybrids that inhibit LSD1 against bladder cancer cells

Jia Liu[1*], Xingwang Zhu[1], Liu Yu[1], Minghuan Mao[1]

[1]Department of Urology, The 4th affiliated hospital of China Medical University, Shenyang, 110032, China.
[*]Corresponding author: Jia Liu (lj18900913068@sina.com)

3,4-Dimethoxy-N-(quinolin-8-yl)benzenesulfonamide (J1)

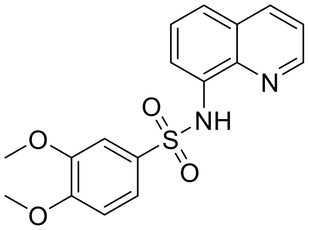

White solid, yield:80%, m.p.:167~169 ºC. ^{1}H NMR (400 MHz, DMSO-d_6) δ 9.84 (s, 1H), 8.88 (dd, J = 4.2, 1.6 Hz, 1H), 8.36 (dd, J = 8.3, 1.6 Hz, 1H), 7.71 (dd, J = 7.6, 1.1 Hz, 1H), 7.68 – 7.63 (m, 1H), 7.59 (dd, J = 8.3, 4.2 Hz, 1H), 7.56 – 7.47 (m, 2H), 7.44 (d, J = 2.2 Hz, 1H), 7.00 (d, J = 8.5 Hz, 1H), 3.74 (s, 3H), 3.69 (s, 3H). ^{13}C NMR (100 MHz, DMSO-d_6) δ 152.34, 149.30, 148.45, 138.67, 136.51, 133.76, 130.76, 128.05, 126.66, 122.77, 122.31, 120.84, 116.35, 110.91, 109.67, 55.72, 55.68. HRMS (m/z): Calcd. $C_{17}H_{17}N_2O_4S$, $[M+H]^+$ m/z: 345.0909, found: 345.0914.

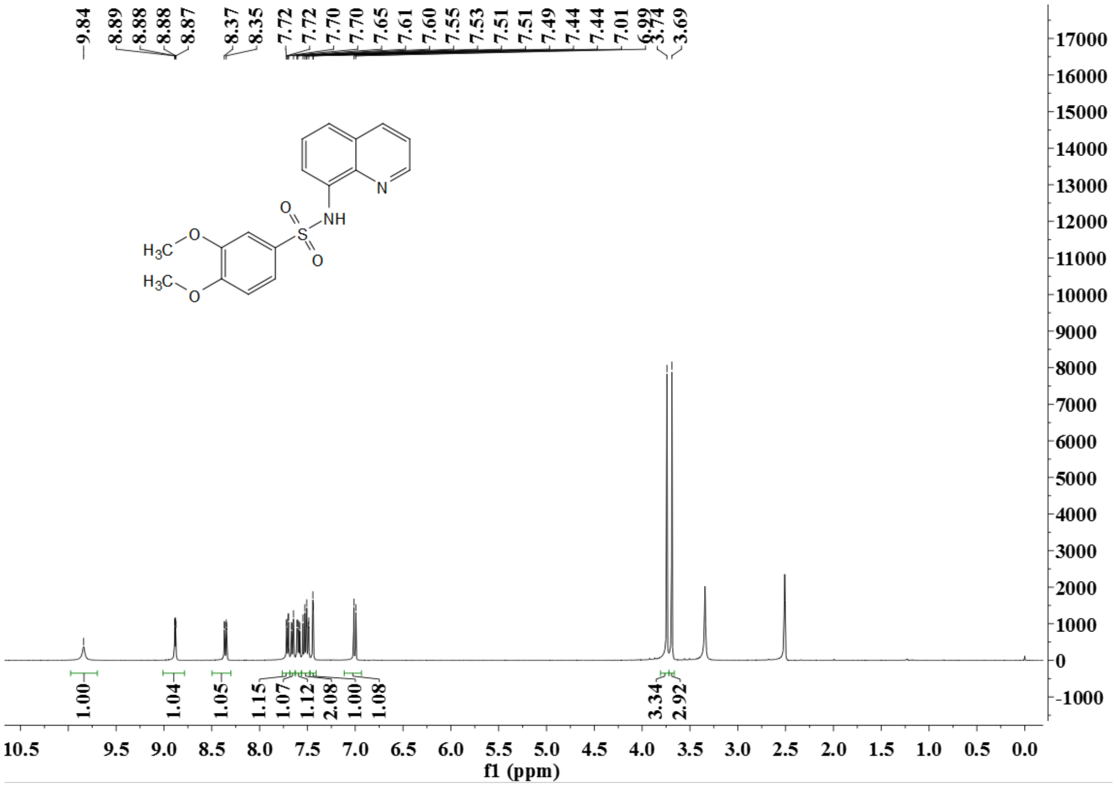

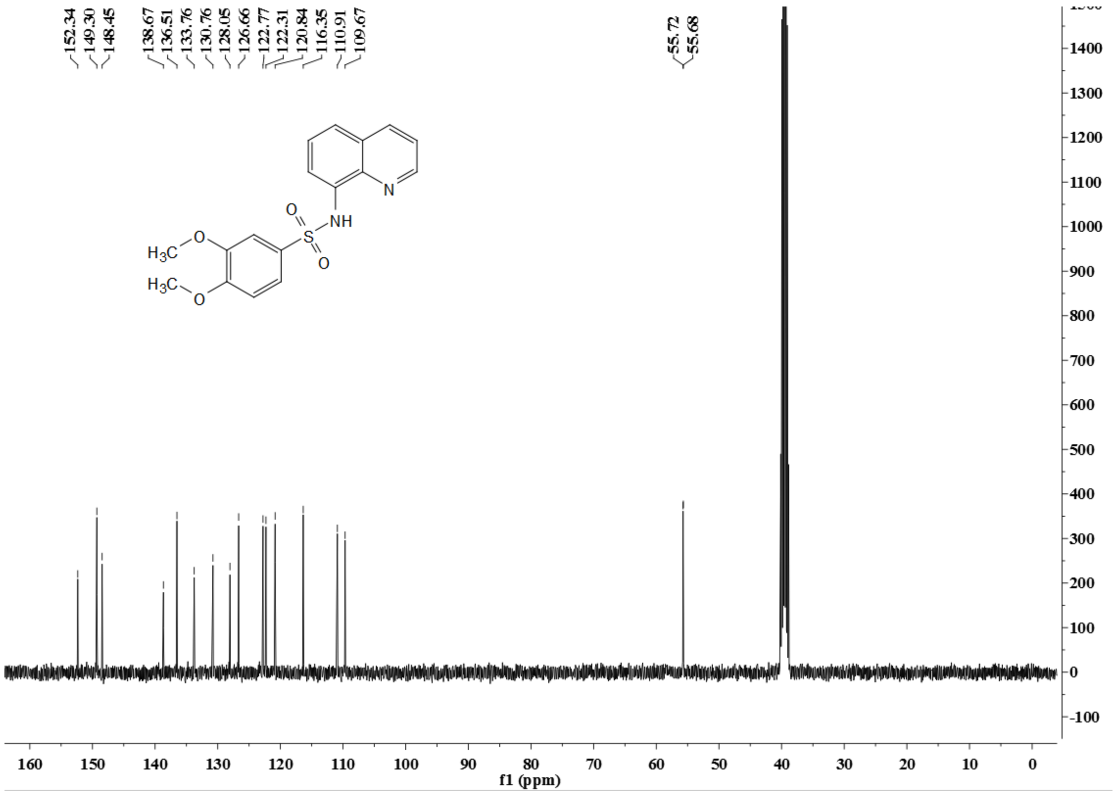

4-Methoxy-N-(quinolin-8-yl)benzenesulfonamide (J2)

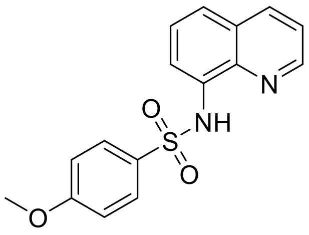

White solid, yield:87%, m.p.:161~163 °C. ^{1}H NMR (400 MHz, DMSO-d_6) δ 9.79 (s, 1H), 8.87 (dd, J = 4.2, 1.6 Hz, 1H), 8.35 (dd, J = 8.3, 1.6 Hz, 1H), 7.98 – 7.80 (m, 2H), 7.77 – 7.41 (m, 4H), 7.09 – 6.91 (m, 2H), 3.74 (s, 3H). ^{13}C NMR (100 MHz, DMSO-d_6) δ 162.62, 149.30, 138.49, 136.51, 133.67, 130.83, 129.17, 128.03, 126.66, 122.67, 122.33, 115.94, 114.27, 55.57. HRMS (m/z): Calcd. $C_{16}H_{15}N_2O_3S$, $[M+H]^+$ m/z: 315.0803, found: 315.0808.

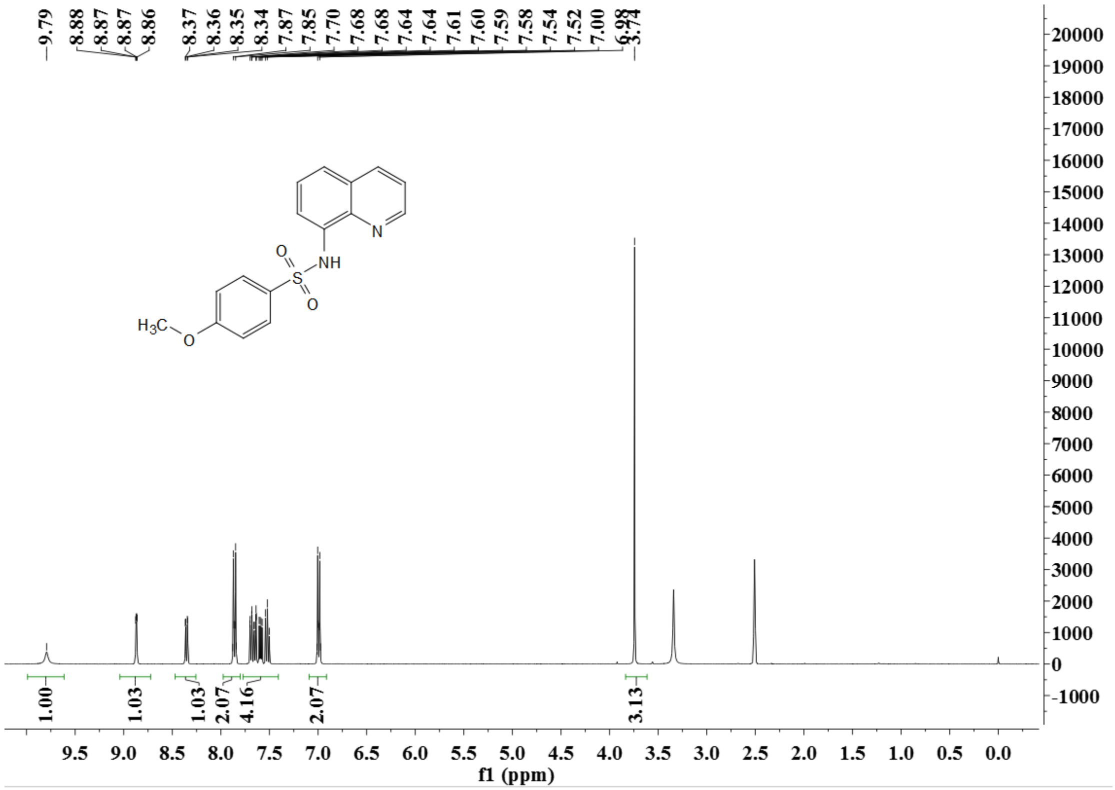

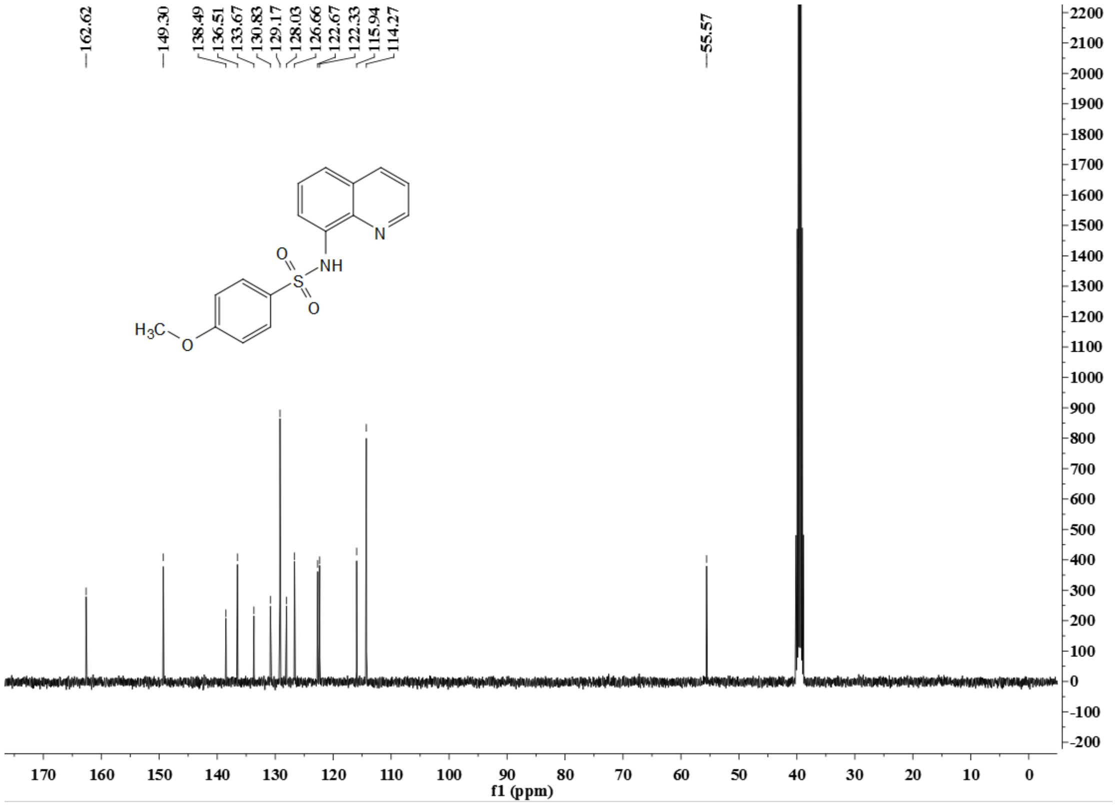

4-(Tert-butyl)-N-(quinolin-8-yl)benzenesulfonamide (J3)

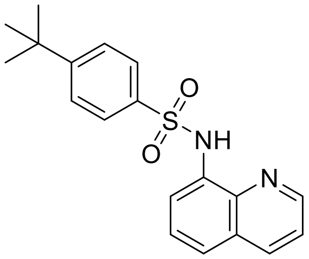

White solid, yield:78%, m.p.:142~144 °C. ^{1}H NMR (400 MHz, DMSO-d_6) δ 9.91 (s, 1H), 8.86 (dd, J = 4.2, 1.6 Hz, 1H), 8.35 (dd, J = 8.3, 1.6 Hz, 1H), 7.92 – 7.80 (m, 2H), 7.71 (dd, J = 7.6, 1.1 Hz, 1H), 7.65 (dd, J = 8.2, 1.0 Hz, 1H), 7.59 (dd, J = 8.3, 4.2 Hz, 1H), 7.55 – 7.46 (m, 3H), 1.20 (s, 9H). ^{13}C NMR (100 MHz, DMSO-d_6) δ 149.34, 139.41, 138.76, 136.48, 133.53, 133.07, 129.10, 128.06, 126.85, 126.62, 123.07, 122.30, 116.77. HRMS (m/z): Calcd. $C_{19}H_{21}N_2O_2S$, $[M+H]^+$ m/z: 341.1324, found: 341.1329.

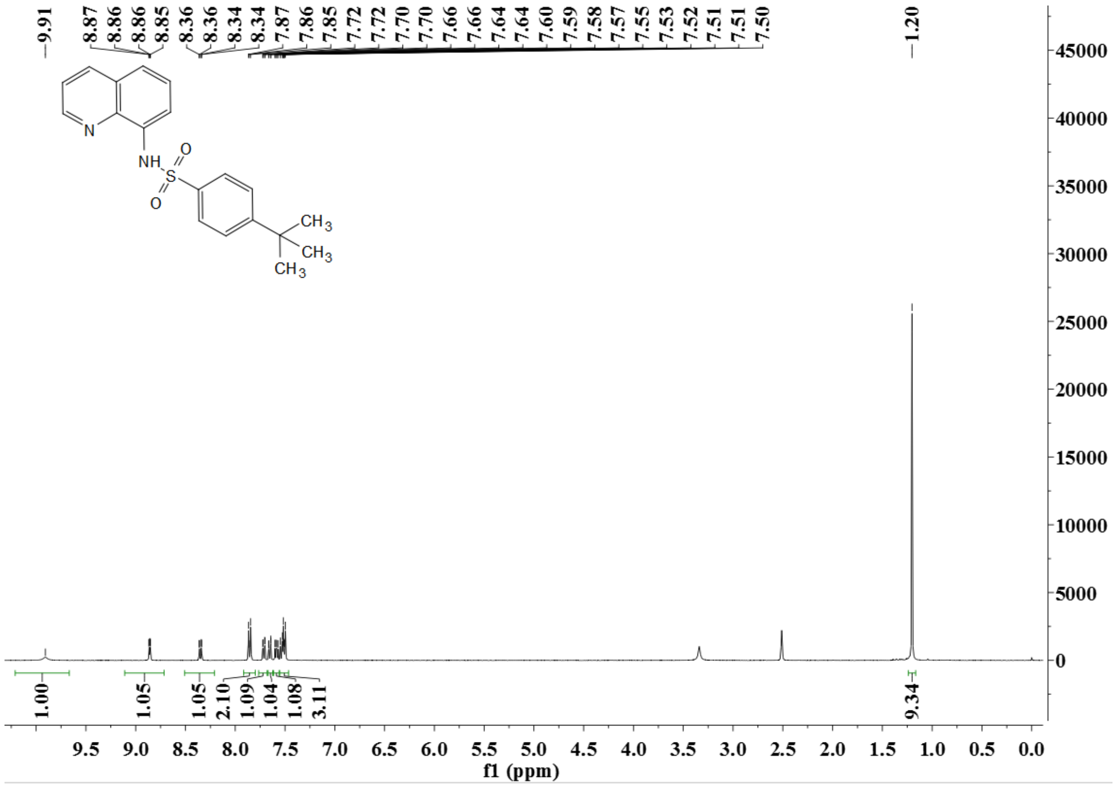

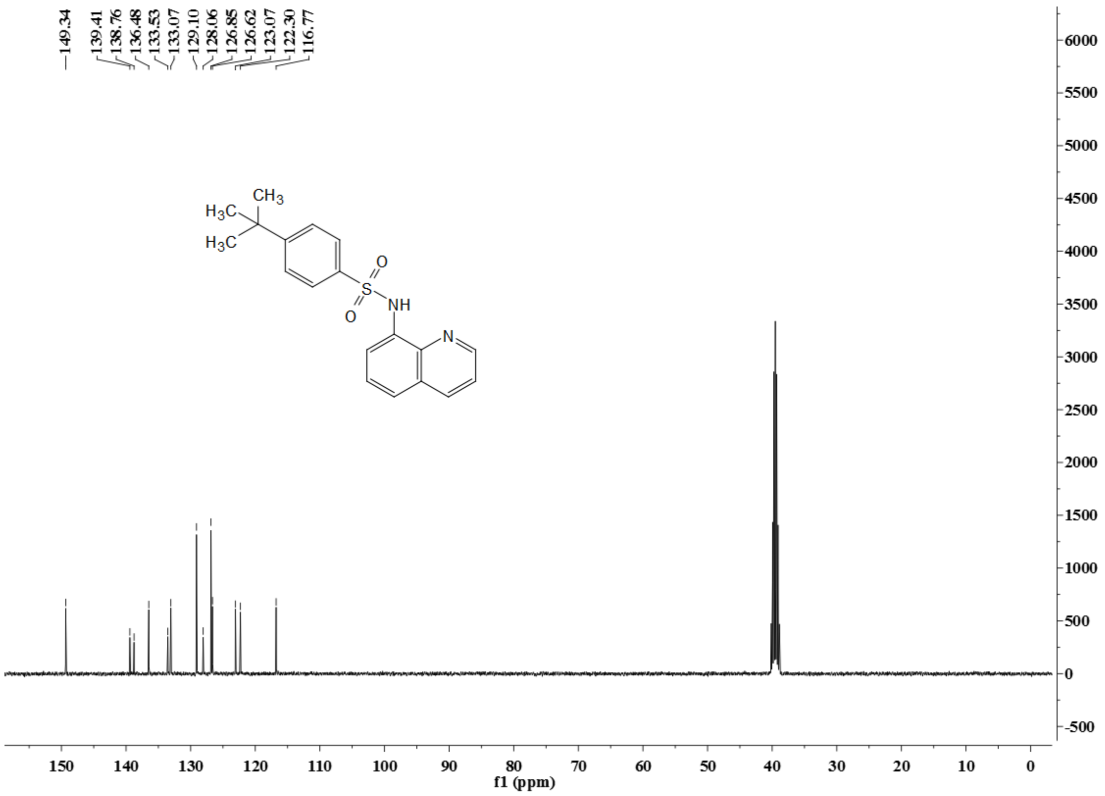

N-(quinolin-8-yl)benzenesulfonamide (J4)

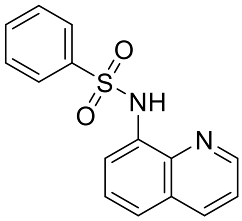

White solid, yield:89%, m.p.:165~167 °C. ^{1}H NMR (400 MHz, DMSO-d_6) δ 9.99 (s, 1H), 8.86 (dd, J = 4.2, 1.6 Hz, 1H), 8.35 (dd, J = 8.3, 1.5 Hz, 1H), 8.05 – 7.86 (m, 2H), 7.79 – 7.62 (m, 2H), 7.62 – 7.39 (m, 5H). ^{13}C NMR (100 MHz, DMSO-d_6) δ 156.15, 149.28, 138.54, 136.61, 136.49, 133.64, 128.07, 126.78, 126.68, 125.98, 122.75, 122.31, 116.02, 34.78, 30.62. HRMS (m/z): Calcd. $C_{15}H_{13}N_2O_2S$, $[M+H]^+$ m/z: 285.0698, found: 285.0699.

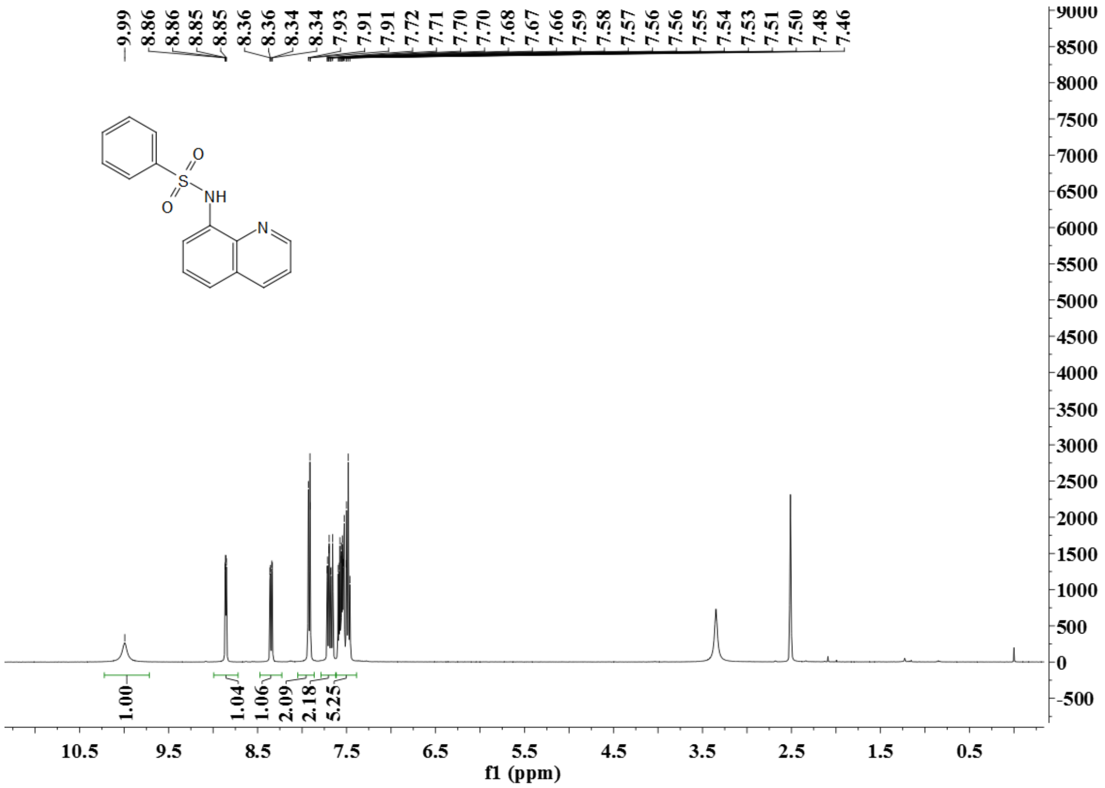

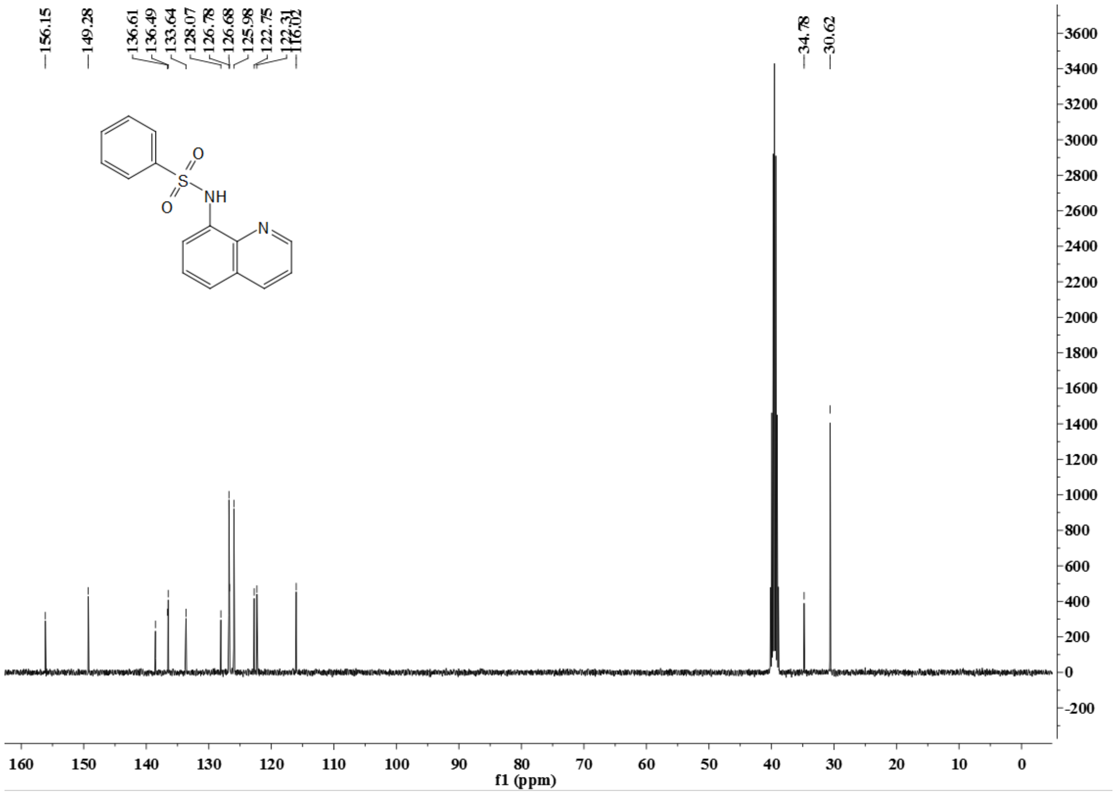

4-Methyl-N-(quinolin-8-yl)benzenesulfonamide (J5)

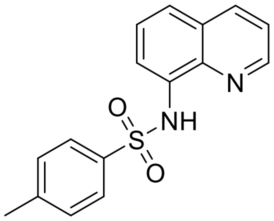

White solid, yield:84%, m.p.:148~150 °C. ^{1}H NMR (400 MHz, DMSO-d_6) δ 9.88 (s, 1H), 8.87 (dd, J = 4.2, 1.5 Hz, 1H), 8.35 (dd, J = 8.3, 1.5 Hz, 1H), 7.81 (d, J = 8.3 Hz, 2H), 7.75 – 7.43 (m, 4H), 7.28 (d, J = 8.1 Hz, 2H), 2.27 (s, 3H). ^{13}C NMR (100 MHz, DMSO-d_6) δ 149.32, 143.56, 138.53, 136.52, 136.46, 133.58, 129.57, 128.04, 126.93, 126.65, 122.80, 122.33, 116.13, 20.86. HRMS (m/z): Calcd. $C_{16}H_{15}N_2O_2S$, $[M+H]^+$ m/z: 299.0854, found: 299.0858.

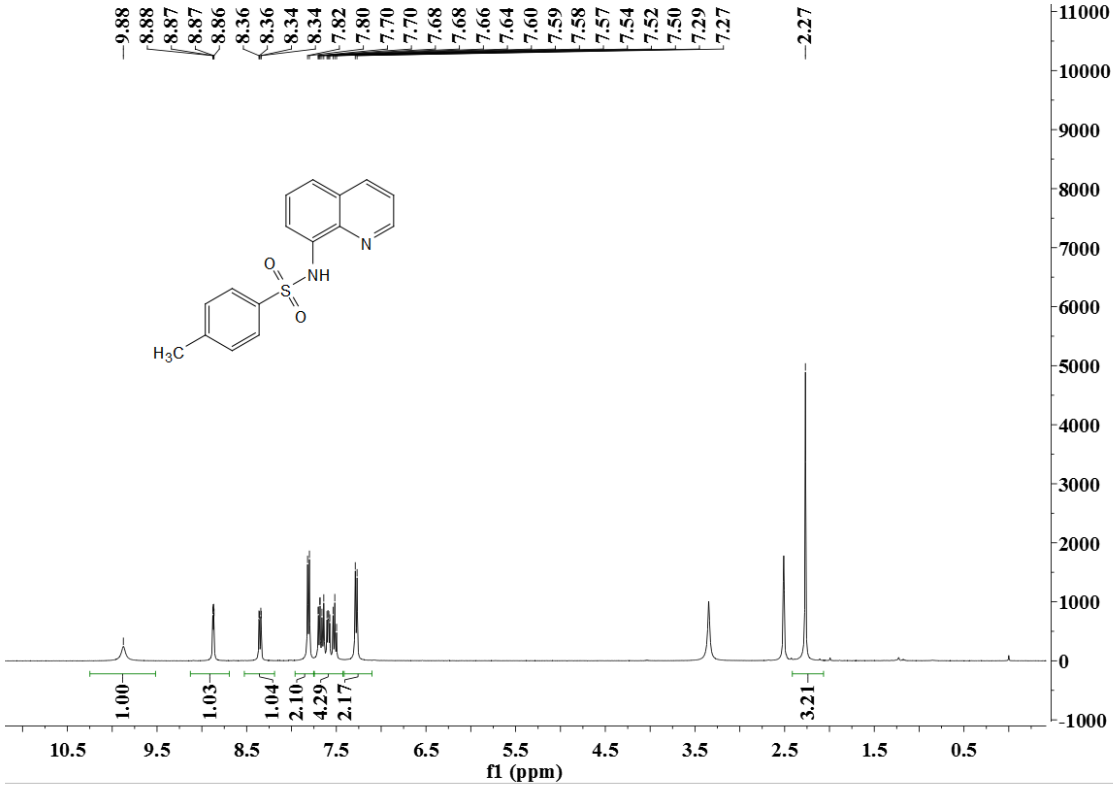

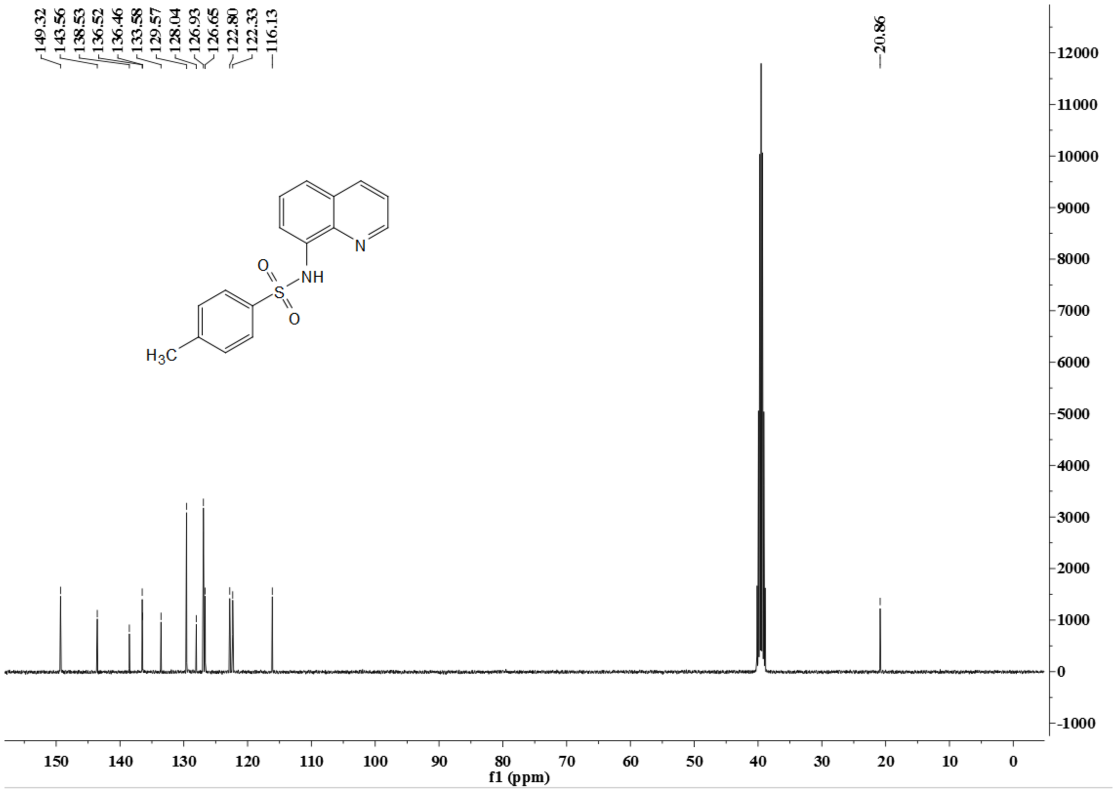

4-Bromo-N-(quinolin-8-yl)benzenesulfonamide (J6)

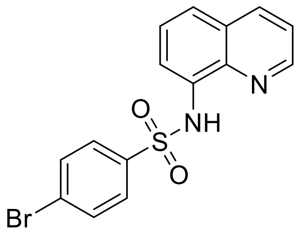

White solid, yield:92%, m.p.:192~194 °C. ^{1}H NMR (400 MHz, DMSO-*d*$_6$) δ 10.19 (s, 1H), 8.85 (dd, *J* = 4.2, 1.6 Hz, 1H), 8.36 (dd, *J* = 8.3, 1.5 Hz, 1H), 7.83 (d, *J* = 8.6 Hz, 2H), 7.73 – 7.67 (m, 4H), 7.63 – 7.41 (m, 2H). ^{13}C NMR (100 MHz, DMSO-*d*$_6$) δ 149.41, 139.09, 138.87, 136.49, 133.35, 132.14, 128.90, 128.14, 126.90, 126.63, 123.51, 122.29, 117.68. HRMS (m/z): Calcd. $C_{15}H_{12}BrN_2O_2S$, $[M+H]^+$ m/z: 362.9803, found: 362.9807.

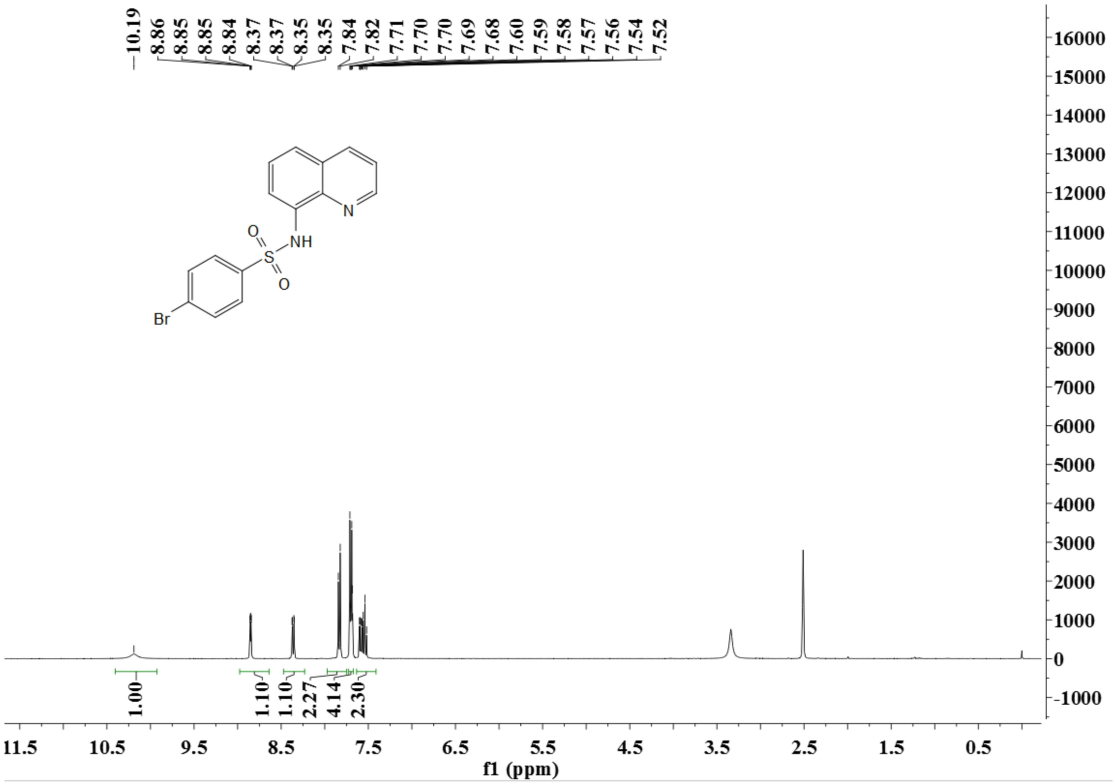

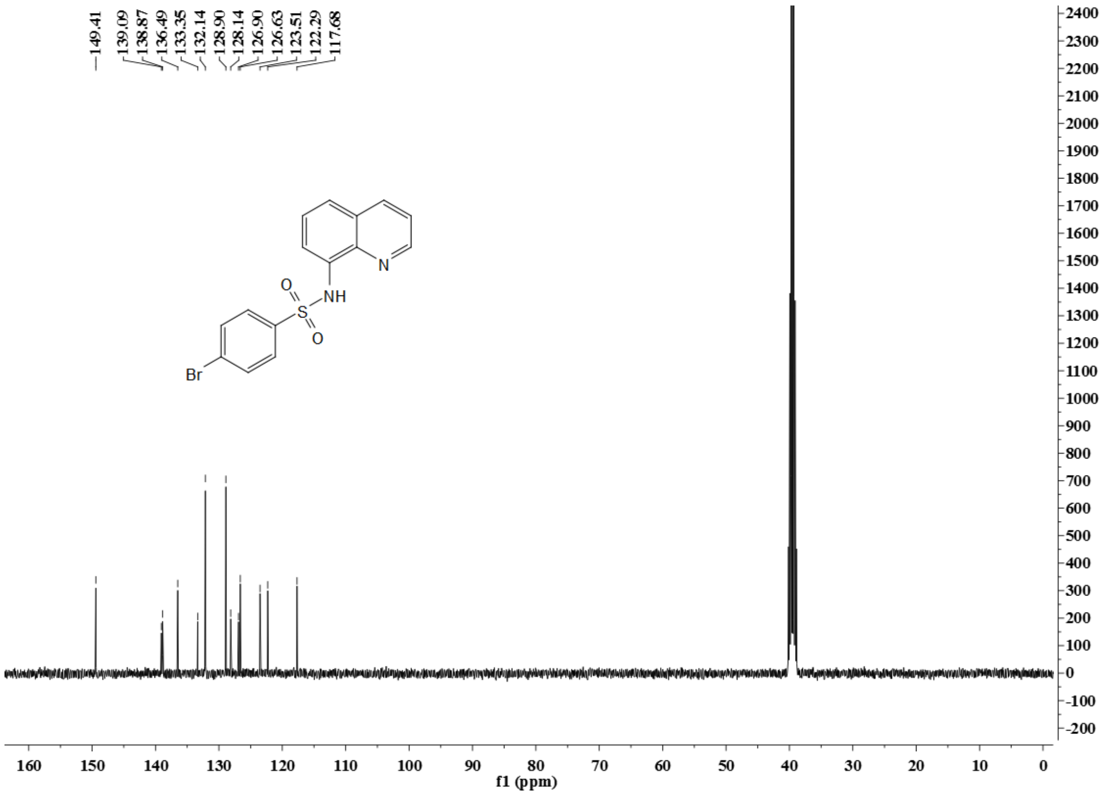

N-(3,4,5-trimethoxyphenyl)quinoline-8-sulfonamide (J7)

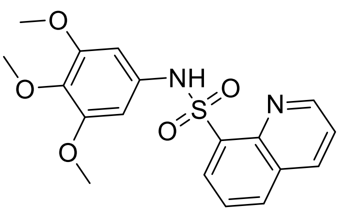

White solid, yield:94%, m.p.:181~183 ^{o}C. ^{1}H NMR (400 MHz, DMSO-d_6) δ 9.87 (s, 1H), 9.16 (dd, J = 4.2, 1.7 Hz, 1H), 8.52 (dd, J = 8.4, 1.7 Hz, 1H), 8.39 (dd, J = 7.3, 1.3 Hz, 1H), 8.27 (dd, J = 8.2, 1.3 Hz, 1H), 7.81 – 7.60 (m, 2H), 6.35 (s, 2H), 3.51 (s, 6H), 3.47 (s, 3H). ^{13}C NMR (100 MHz, DMSO-d_6) δ 152.59, 151.40, 142.72, 136.98, 135.12, 134.23, 133.92, 133.57, 132.25, 128.34, 125.63, 122.58, 98.02, 59.91, 55.56. HRMS (m/z): Calcd. $C_{18}H_{19}N_2O_5S$, $[M+H]^+$ m/z: 375.1015, found: 375.1019.

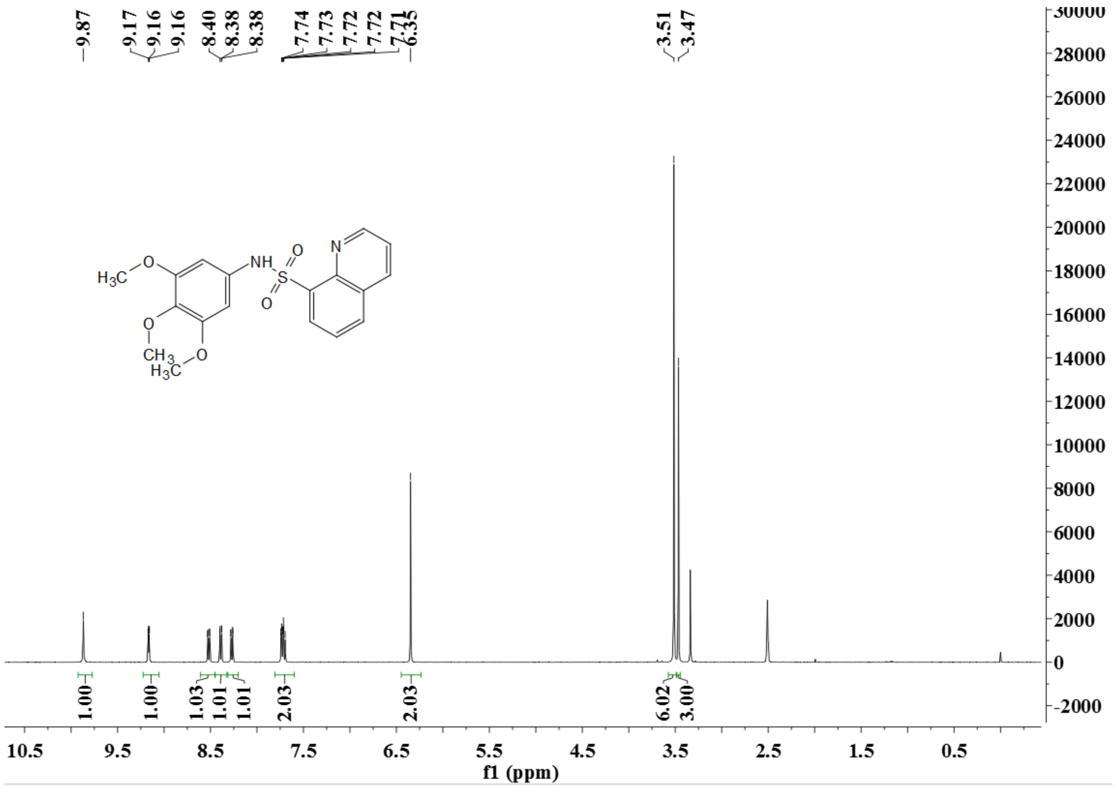

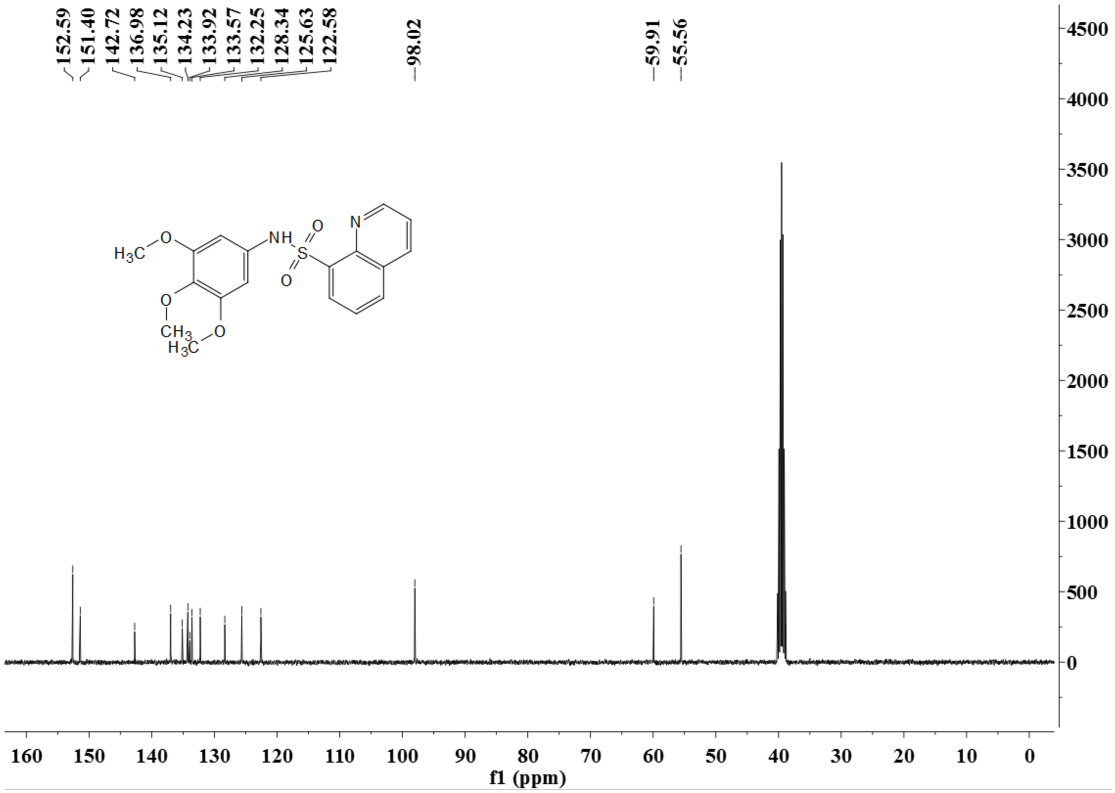

N-(2-bromoethyl)-N-(3,4,5-trimethoxyphenyl)quinoline-8-sulfonamide (K1)

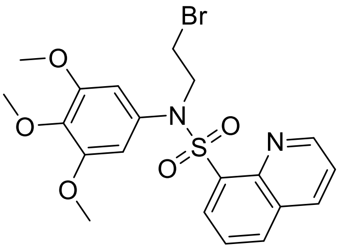

White solid, yield:72%, m.p.:158~160 °C. ^{1}H NMR (400 MHz, $CDCl_3$) δ 9.11 (dd, *J* = 4.2, 1.8 Hz, 1H), 8.21 (dd, *J* = 8.6, 1.5 Hz, 2H), 7.94 (dd, *J* = 8.2, 1.3 Hz, 1H), 7.66 – 7.36 (m, 2H), 6.09 (s, 2H), 4.49 (t, *J* = 7.5 Hz, 2H), 3.67 (s, 3H), 3.48 (t, *J* = 7.5 Hz, 2H), 3.40 (s, 6H). ^{13}C NMR (100 MHz, $CDCl_3$) δ 152.07, 150.20, 143.11, 136.85, 135.75, 135.63, 133.29, 133.13, 132.51, 127.65, 124.69, 121.11, 105.47, 59.75, 54.85, 53.99, 29.17. HRMS (m/z): Calcd. $C_{20}H_{22}BrN_2O_5S$, $[M+H]^+$ m/z: 481.0433, found: 481.0439.

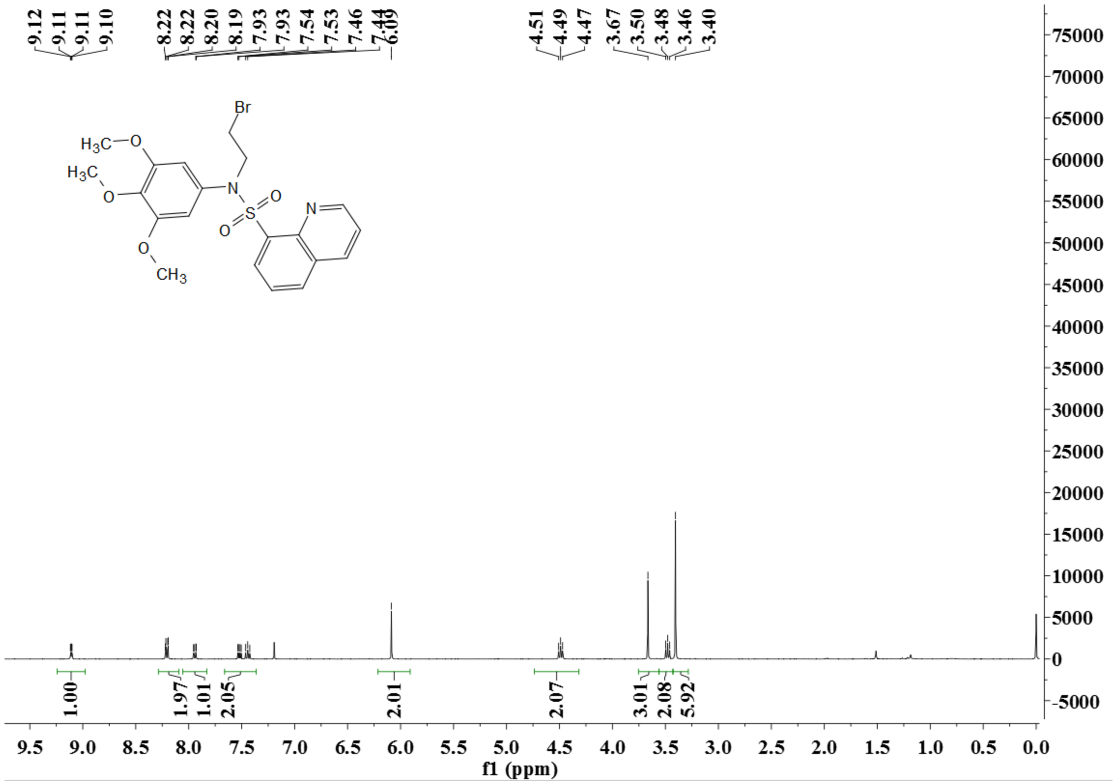

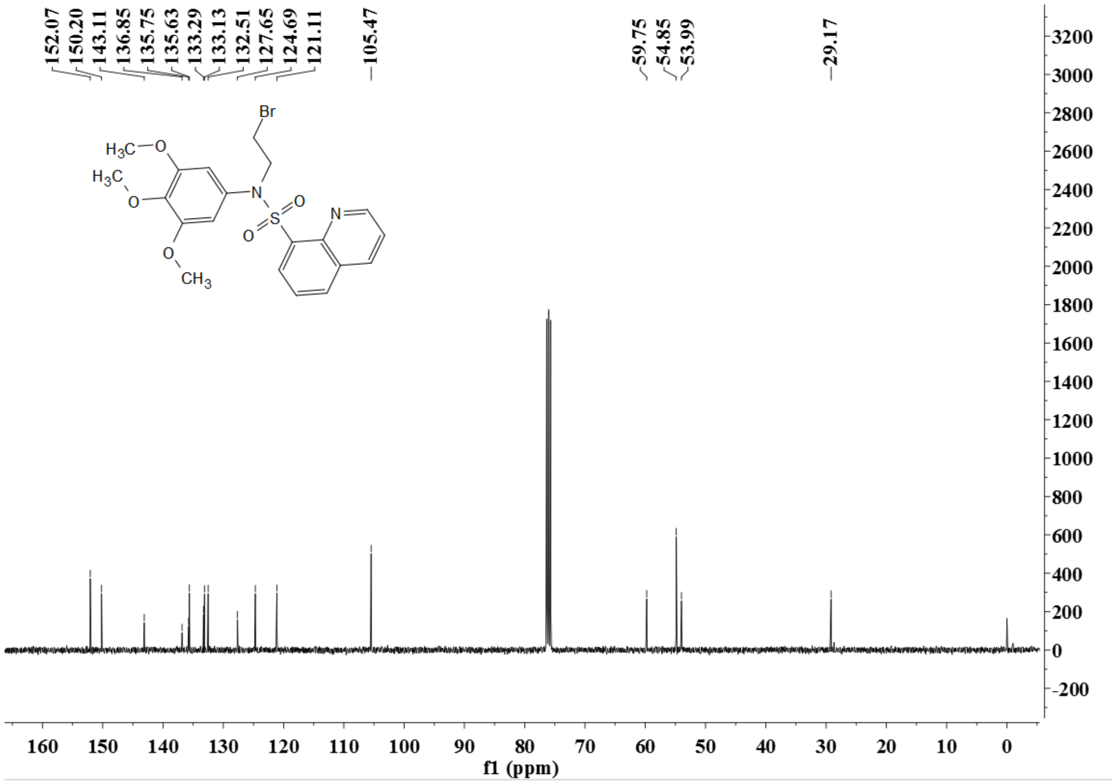

N-(3-bromopropyl)-N-(3,4,5-trimethoxyphenyl)quinoline-8-sulfonamide (K2)

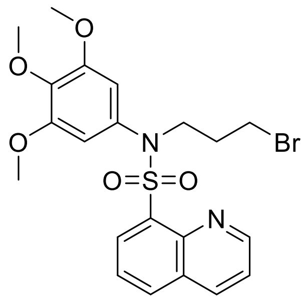

White solid, yield:81%, m.p.:129~130 ºC. ^{1}H NMR (400 MHz, $CDCl_3$) δ 9.09 (dd, *J* = 4.2, 1.7 Hz, 1H), 8.20 (td, *J* = 8.6, 1.5 Hz, 2H), 7.93 (dd, *J* = 8.2, 1.2 Hz, 1H), 7.62 – 7.32 (m, 2H), 6.12 (s, 2H), 4.22 (t, *J* = 6.8 Hz, 2H), 3.67 (s, 3H), 3.47 (t, *J* = 6.8 Hz, 2H), 3.42 (d, *J* = 8.3 Hz, 6H), 2.09 (p, *J* = 6.8 Hz, 2H). ^{13}C NMR (100 MHz, $CDCl_3$) δ 152.00, 150.12, 143.22, 136.55, 135.78, 135.56, 133.61, 133.14, 132.46, 127.67, 124.63, 121.03, 105.19, 59.77, 54.89, 51.02, 31.75, 29.45. HRMS (m/z): Calcd. $C_{21}H_{24}BrN_2O_5S$, $[M+H]^+$ m/z: 495.0589, found: 495.0593.

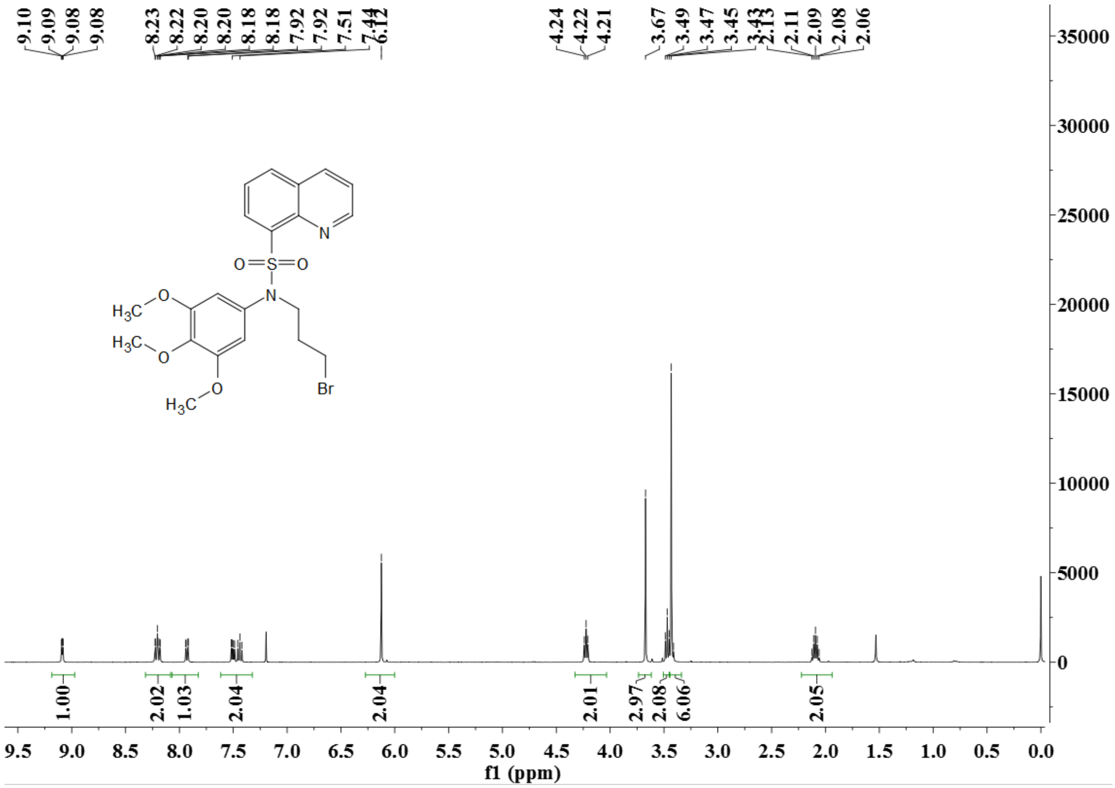

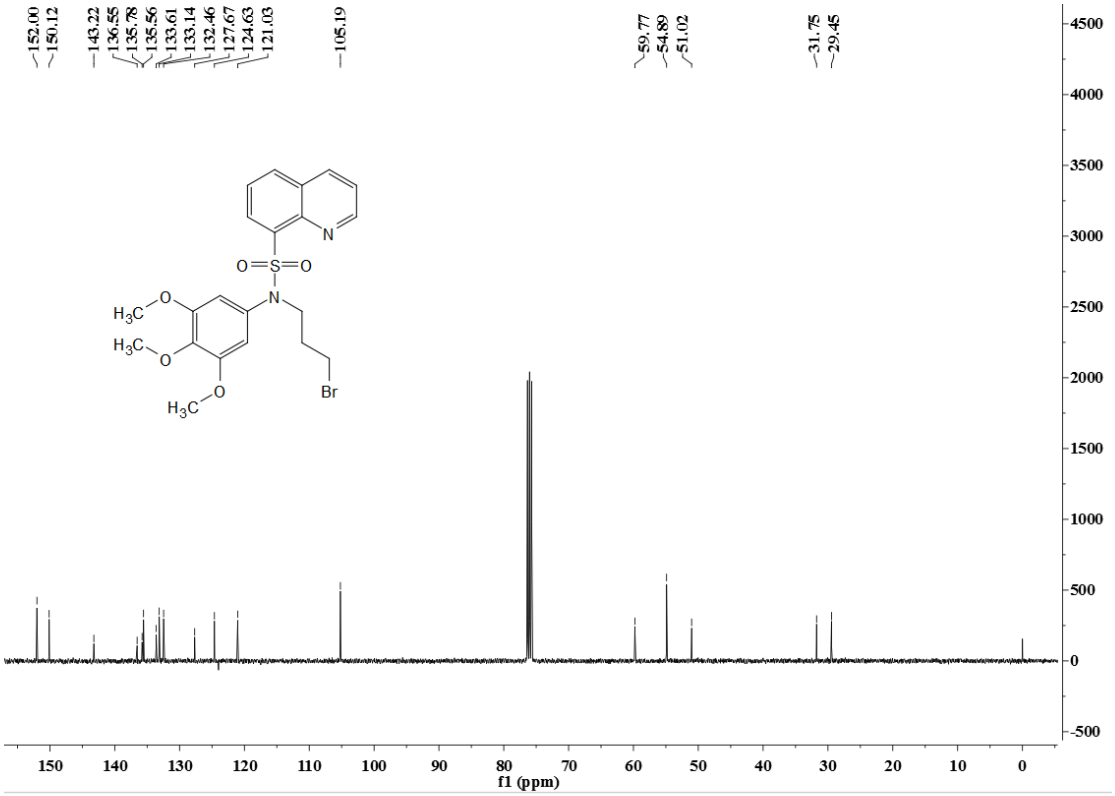

N-(4-bromobutyl)-N-(3,4,5-trimethoxyphenyl)quinoline-8-sulfonamide (K3)

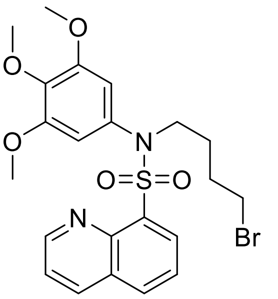

White solid, yield:96%, m.p.:103~104 °C. ^{1}H NMR (400 MHz, $CDCl_3$) δ 9.08 (dd, *J* = 4.2, 1.8 Hz, 1H), 8.19 (ddd, *J* = 6.0, 3.9, 1.6 Hz, 2H), 7.92 (dd, *J* = 8.2, 1.3 Hz, 1H), 7.60 – 7.31 (m, 2H), 6.08 (s, 2H), 4.12 (t, *J* = 6.8 Hz, 2H), 3.67 (s, 3H), 3.53 – 3.33 (m, 8H), 2.01 (dt, *J* = 14.4, 6.6 Hz, 2H), 1.64 (dt, *J* = 14.1, 6.9 Hz, 2H). ^{13}C NMR (100 MHz, $CDCl_3$) δ 151.95, 150.08, 143.25, 136.54, 136.01, 135.52, 133.50, 133.06, 132.31, 127.64, 124.63, 120.98, 105.48, 59.76, 54.87, 51.59, 32.67, 28.66, 26.64. HRMS (m/z): Calcd. $C_{22}H_{26}BrN_2O_5S$, $[M+H]^+$ m/z: 509.0746, found: 509.0748.

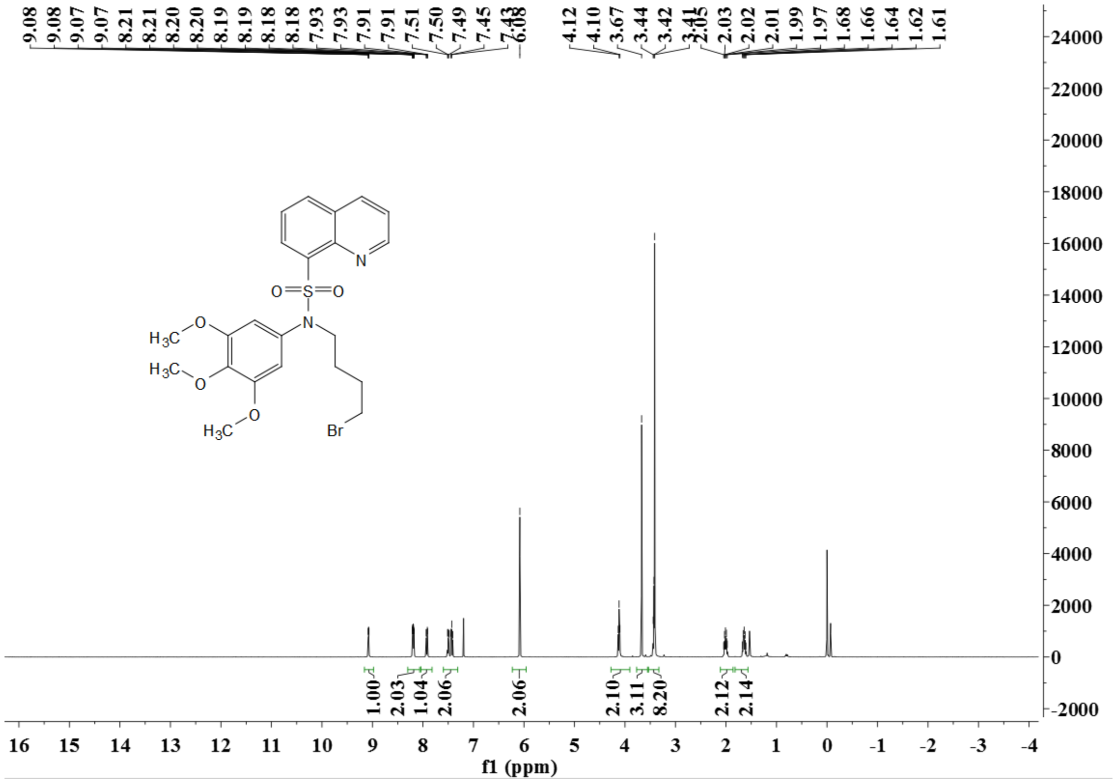

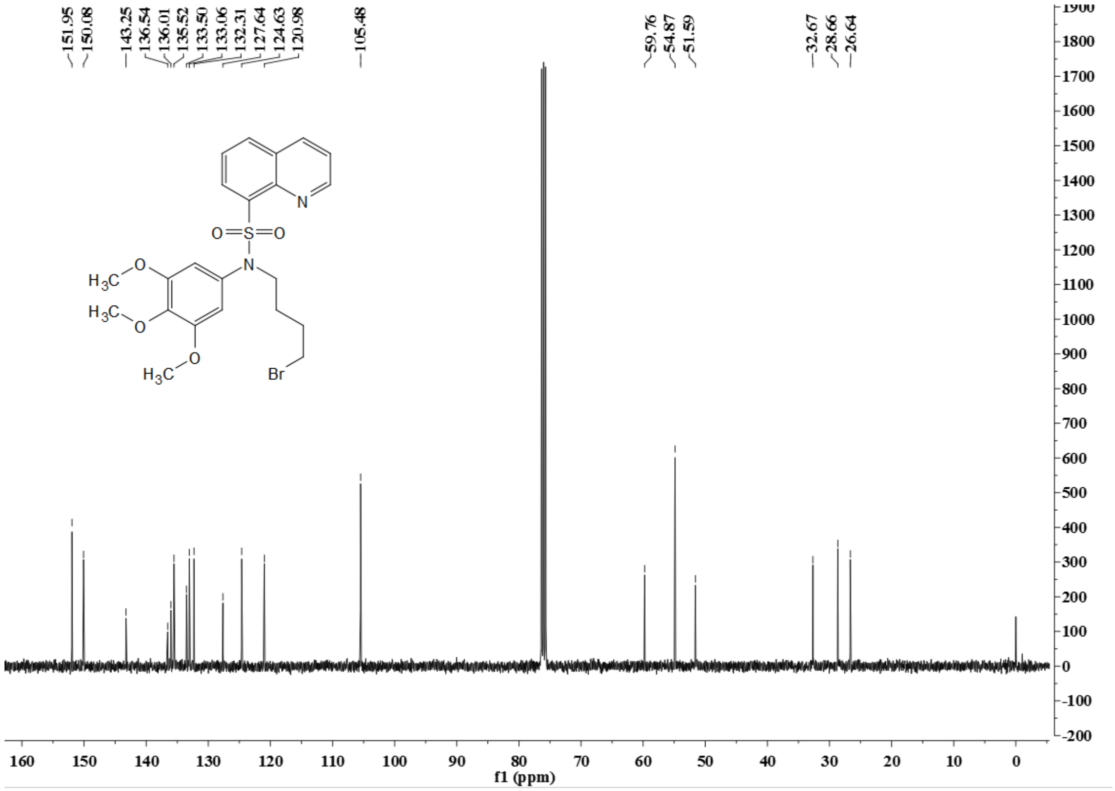

Tert-butyl-4-(((2-(N-(3,4,5-trimethoxyphenyl)quinoline-8-sulfonamido)ethyl)thio)carbonothioyl)piperazine-1-carboxylate (L1)

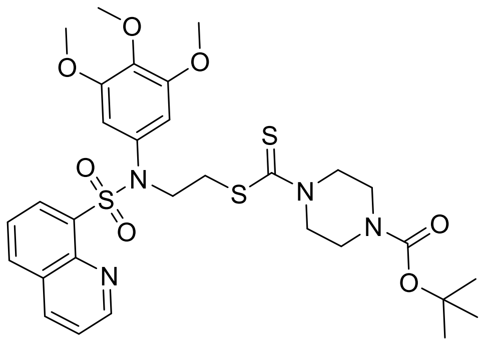

White solid, yield:62%, m.p.:121~123 °C. ^{1}H NMR (400 MHz, $CDCl_3$) δ 9.07 (dd, J = 4.2, 1.8 Hz, 1H), 8.21 (ddd, J = 10.1, 7.9, 1.5 Hz, 2H), 7.93 (dd, J = 8.2, 1.3 Hz, 1H), 7.58 – 7.34 (m, 2H), 6.17 (s, 2H), 4.41 (t, J = 7.0 Hz, 2H), 4.34 – 4.06 (m, 2H), 4.04 – 3.76 (m, 2H), 3.67 (s, 3H), 3.52 (t, J = 7.0 Hz, 2H), 3.47 (dd, J = 10.0, 5.0 Hz, 4H), 3.44 (s, 6H), 1.41 (s, 9H). ^{13}C NMR (100 MHz, $CDCl_3$) δ 195.89, 153.45, 151.90, 150.18, 143.16, 136.51, 135.93, 135.52, 133.33, 133.03, 132.43, 127.64, 124.62, 121.01, 105.28, 79.58, 59.77, 54.91, 50.77, 35.22, 27.35. HRMS (m/z): Calcd. $C_{30}H_{39}N_4O_7S_3$, $[M+H]^+$ m/z: 663.1981, found: 663.1987.

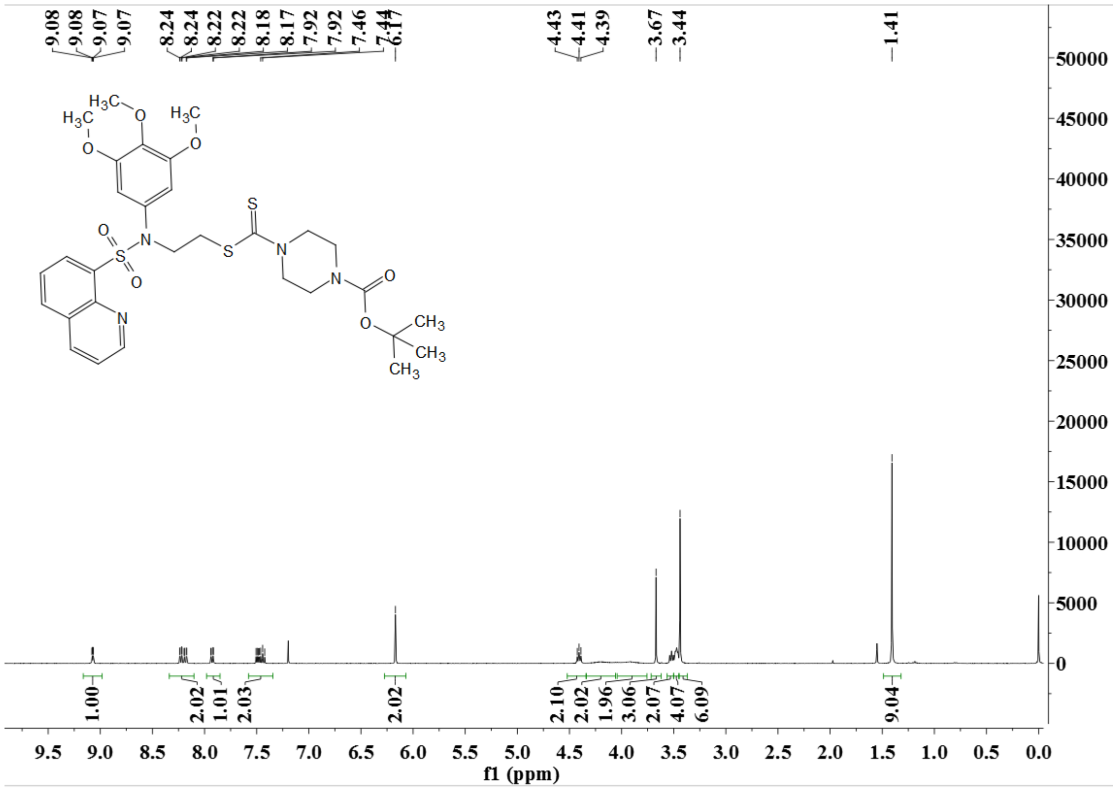

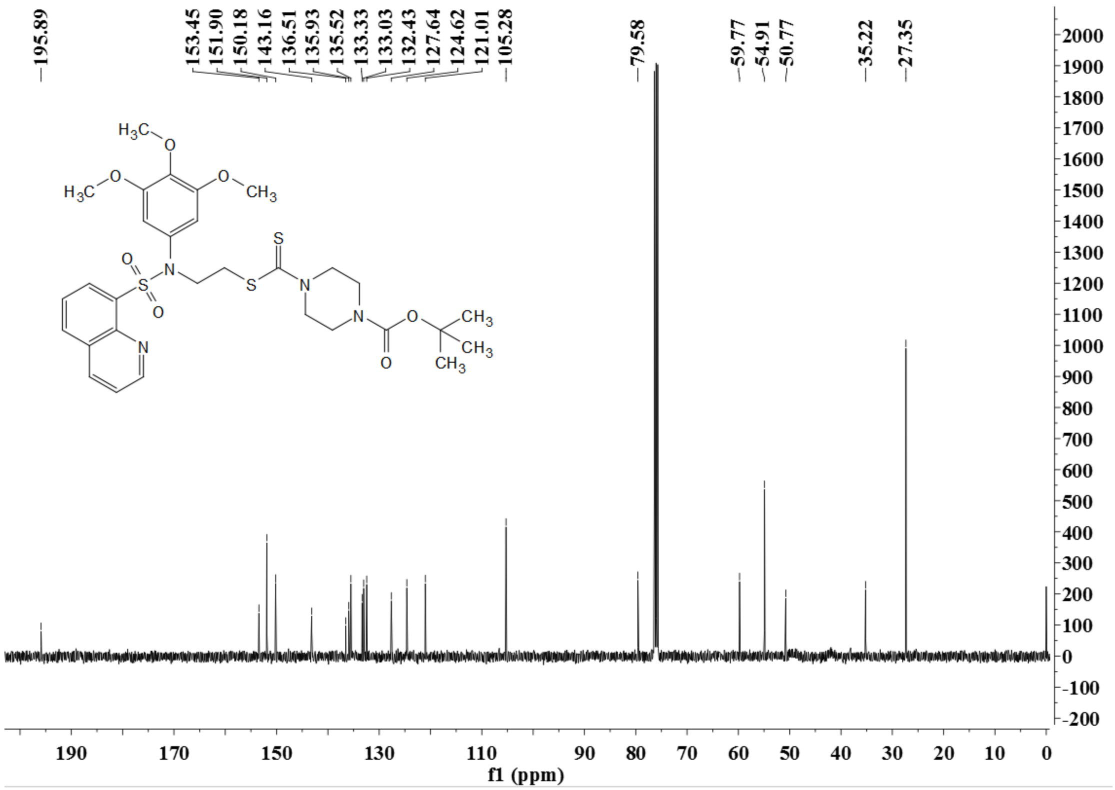

Tert-butyl-4-(((3-(N-(3,4,5-trimethoxyphenyl)quinoline-8-sulfonamido)propyl)thio)carbonothioyl)piperazine-1-carboxylate (L2)

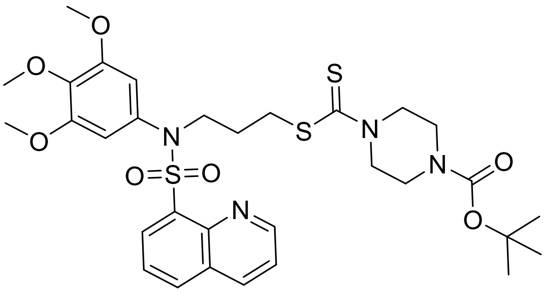

White solid, yield:91%, m.p.:130~132 °C. ^{1}H NMR (400 MHz, $CDCl_3$) δ 9.09 (dd, *J* = 4.2, 1.8 Hz, 1H), 8.35 – 8.06 (m, 2H), 7.92 (dd, *J* = 8.2, 1.3 Hz, 1H), 7.63 – 7.35 (m, 2H), 6.13 (s, 2H), 4.20 (t, *J* = 6.7 Hz, 4H), 3.91 (s, 2H), 3.67 (s, 3H), 3.51 – 3.44 (m, 4H), 3.45 – 3.36 (m, 8H), 2.00 – 1.85 (m, 2H), 1.40 (s, 9H). ^{13}C NMR (100 MHz, $CDCl_3$) δ 196.61, 153.47, 151.94, 150.17, 143.24, 136.50, 135.97, 135.51, 133.57, 133.07, 132.34, 127.64, 124.61, 121.01, 105.45, 79.56, 59.75, 54.90, 51.54, 33.18, 27.76, 27.35. HRMS (m/z): Calcd. $C_{31}H_{41}N_4O_7S_3$, $[M+H]^+$ m/z: 677.2137, found: 677.2139.

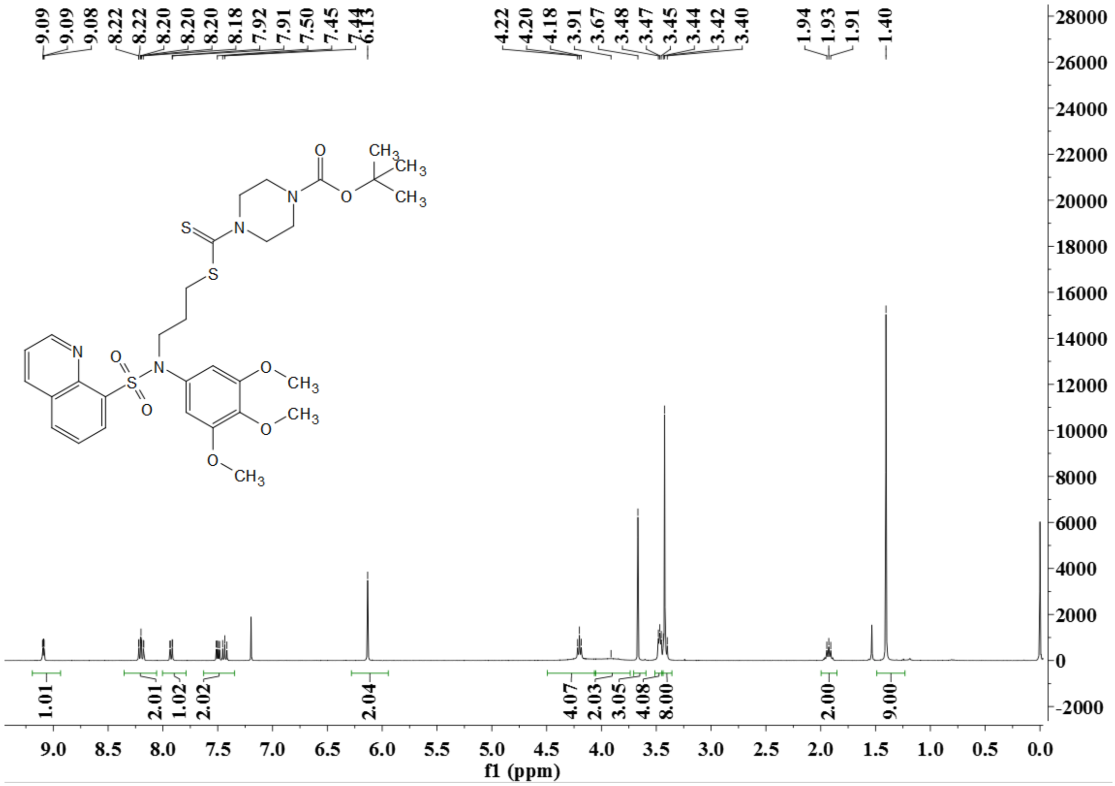

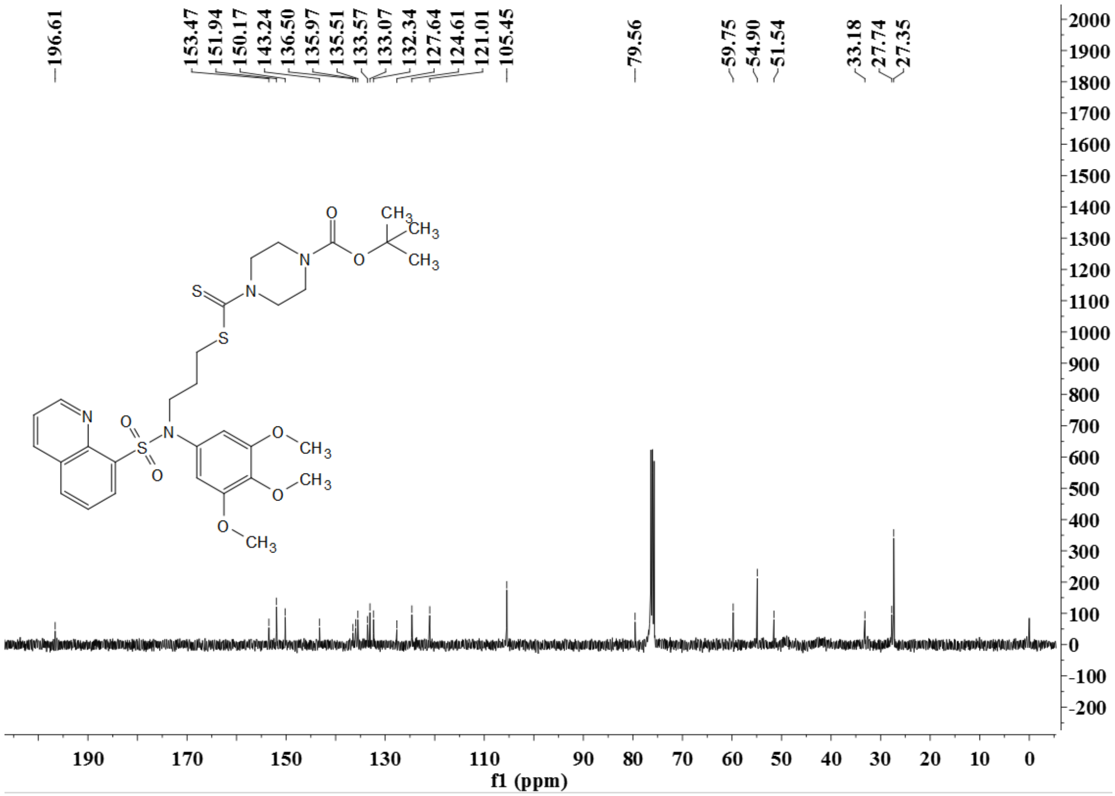

2-(N-(3,4,5-trimethoxyphenyl)quinoline-8-sulfonamido)ethyl-4-(2-hydroxyethyl)piperazine-1-carbodithioate (L3)

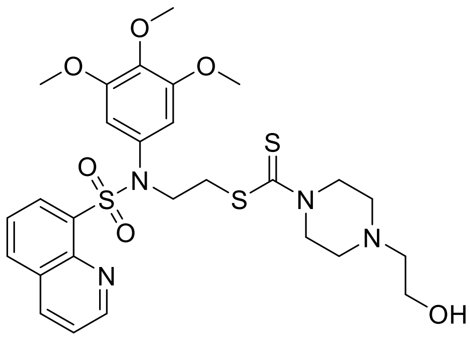

White solid, yield:89%, m.p.:122~124 °C. ^{1}H NMR (400 MHz, DMSO-d_6) δ 9.16 (dd, J = 4.1, 1.5 Hz, 1H), 8.57 (dd, J = 8.4, 1.4 Hz, 1H), 8.29 (d, J = 7.5 Hz, 1H), 8.21 (d, J = 7.3 Hz, 1H), 7.75 (dd, J = 8.3, 4.2 Hz, 1H), 7.65 (t, J = 7.8 Hz, 1H), 6.24 (s, 2H), 4.48 (t, J = 5.3 Hz, 1H), 4.35 (t, J = 6.8 Hz, 2H), 4.19 (s, 2H), 3.86 (s, 2H), 3.55 (d, J = 6.5 Hz, 3H), 3.52 (dd, J = 11.5, 5.9 Hz, 2H), 3.42 (s, 6H), 3.39 (d, J = 6.8 Hz, 2H), 2.50 – 2.46 (m, 4H), 2.43 (t, J = 6.1 Hz, 2H). ^{13}C NMR (100 MHz, DMSO-d_6) δ 194.23, 152.42, 151.54, 143.26, 137.00, 136.75, 136.02, 134.24, 133.97, 133.38, 128.49, 125.67, 122.56, 106.00, 59.95, 59.45, 58.46, 55.55, 52.47, 50.94, 35.54. HRMS (m/z): Calcd. $C_{27}H_{35}N_4O_6S_3$, $[M+H]^+$ m/z: 607.1719, found: 607.1723.

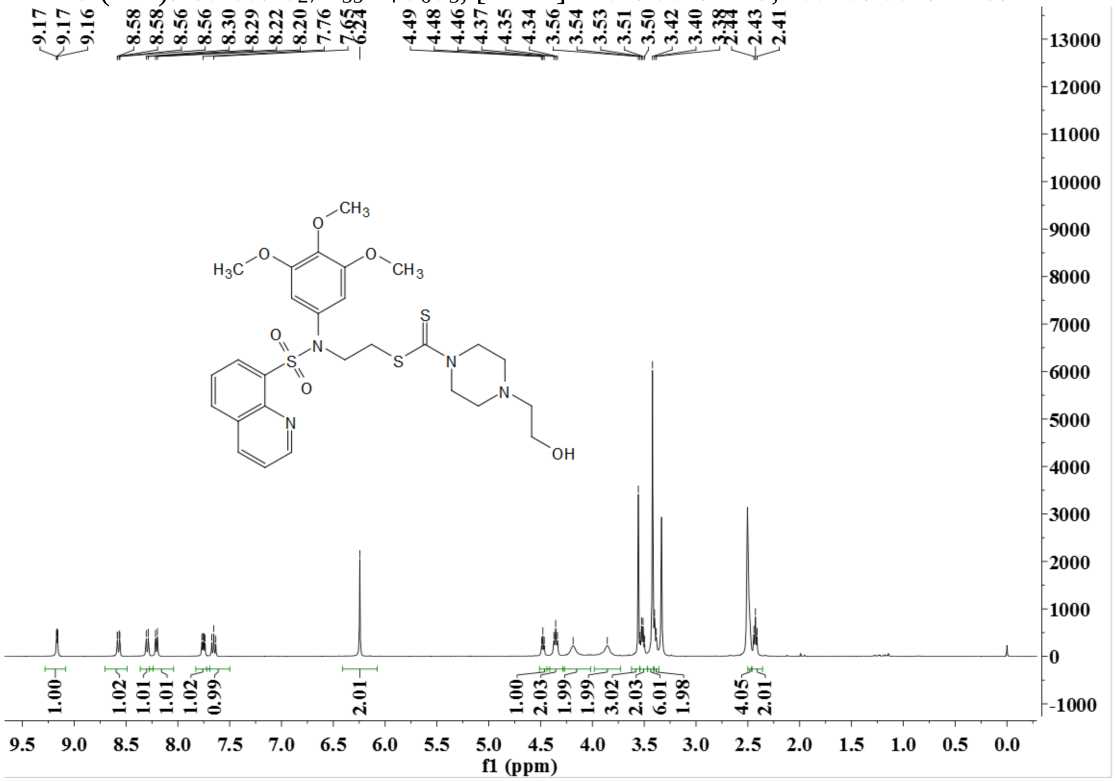

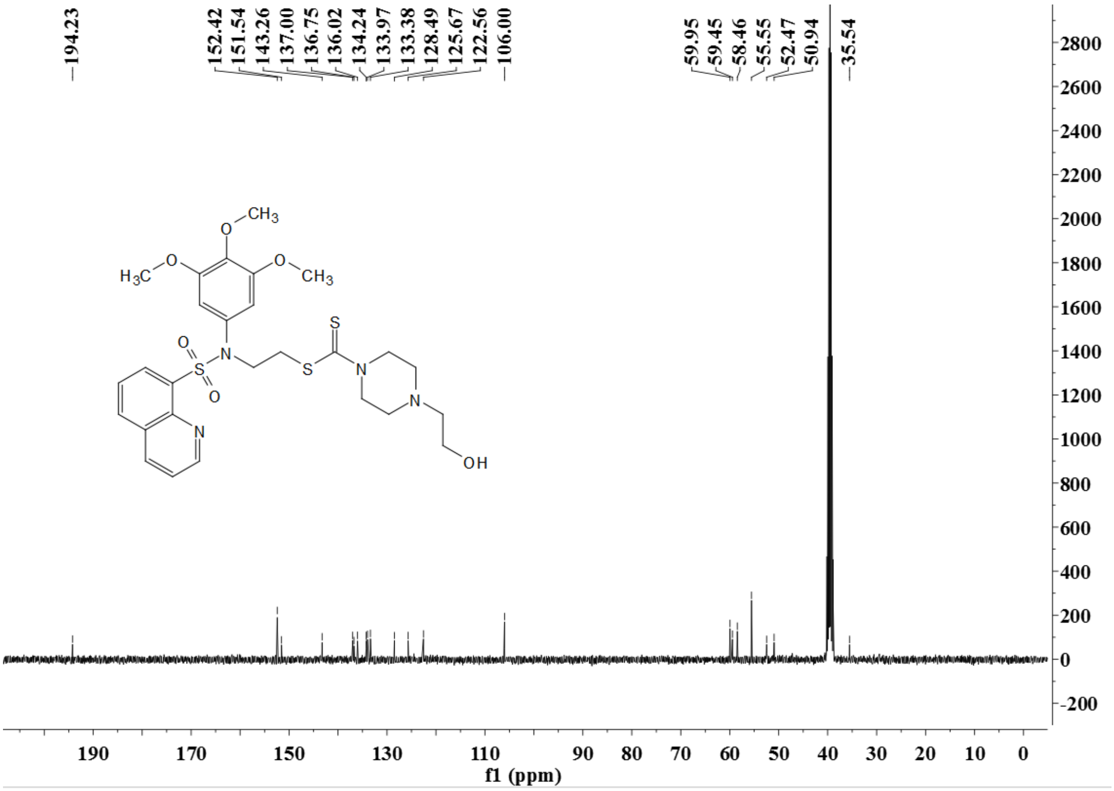

3-(N-(3,4,5-trimethoxyphenyl)quinoline-8-sulfonamido)propyl-4-(2-hydroxyethyl)piperazine-1-carbodithioate (L4)

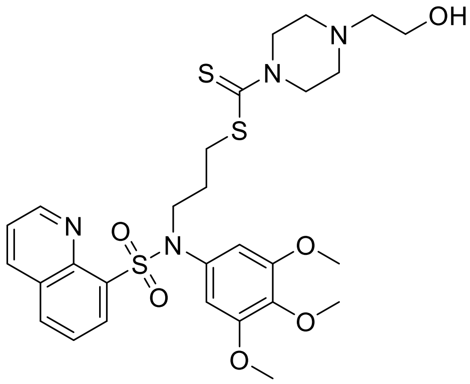

White solid, yield:85%, m.p.:106~107 ºC. ^{1}H NMR (400 MHz, $CDCl_3$) δ 9.10 (d, *J* = 2.1 Hz, 1H), 8.20 (dd, *J* = 6.8, 3.9 Hz, 2H), 7.93 (d, *J* = 8.1 Hz, 1H), 7.65 – 7.34 (m, 2H), 6.12 (s, 2H), 4.33 (s, 2H), 4.20 (t, *J* = 6.6 Hz, 2H), 3.96 (s, 2H), 3.67 (s, 3H), 3.66 – 3.57 (m, 2H), 3.42 (s, 8H), 2.73 – 2.49 (m, 6H), 1.93 (dd, *J* = 13.7, 6.8 Hz, 2H). ^{13}C NMR (100 MHz, $CDCl_3$) δ 196.25, 151.89, 150.19, 143.20, 136.34, 135.86, 135.53, 133.54, 133.08, 132.36, 127.61, 124.62, 121.02, 105.31, 59.76, 58.16, 56.69, 54.85, 51.52, 51.28, 33.21, 27.73. HRMS (m/z): Calcd. $C_{28}H_{37}N_4O_6S_3$, $[M+H]^+$ m/z: 621.1875, found: 621.1879.

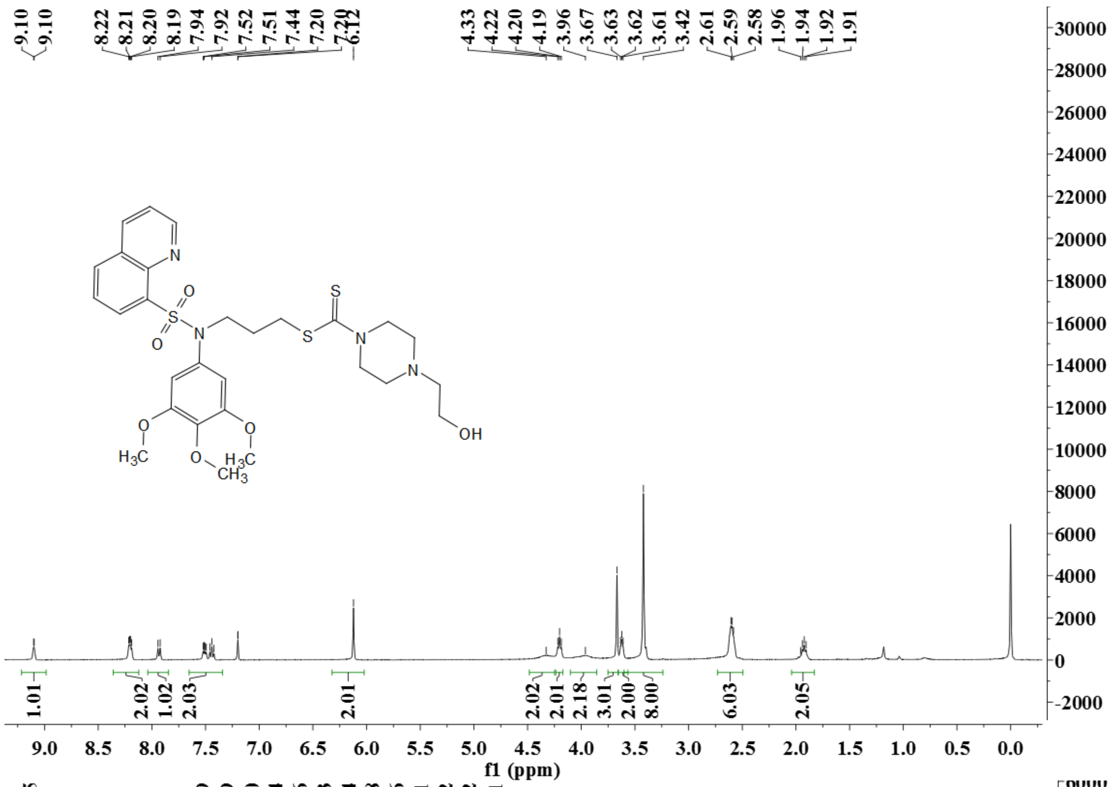

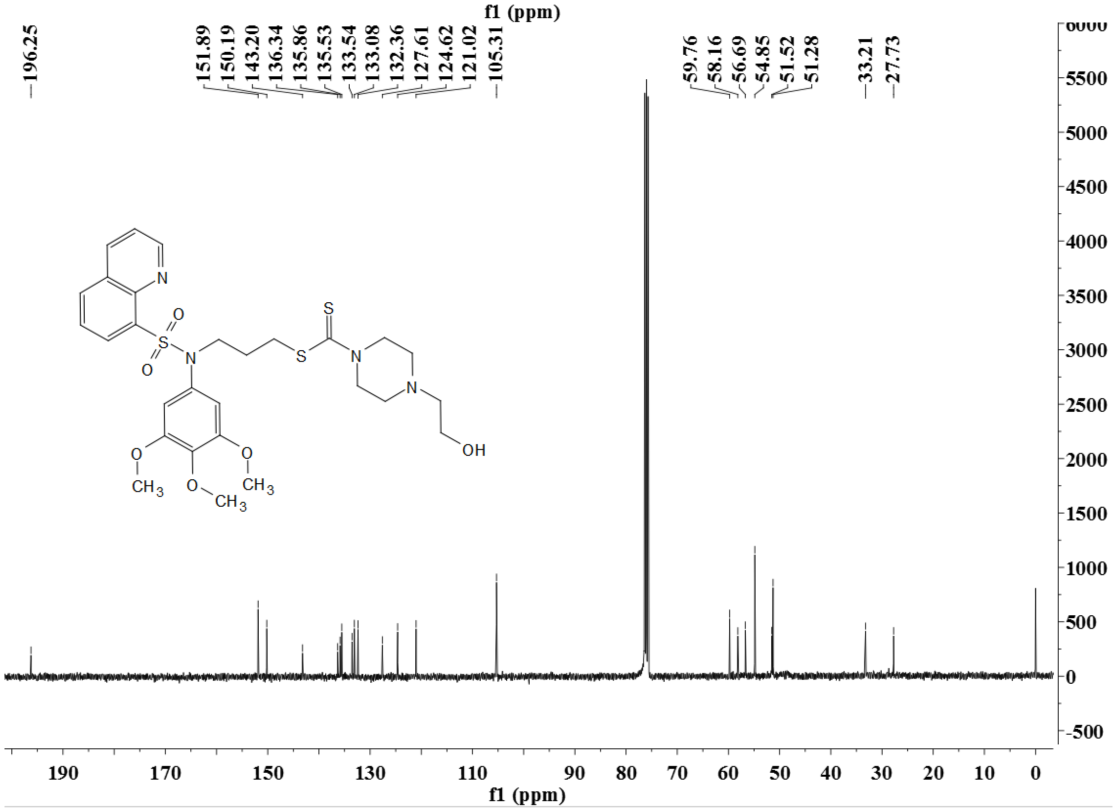

4-(N-(3,4,5-trimethoxyphenyl)quinoline-8-sulfonamido)butyl-4-ethylpiperazine-1-carbodithioate (L5)

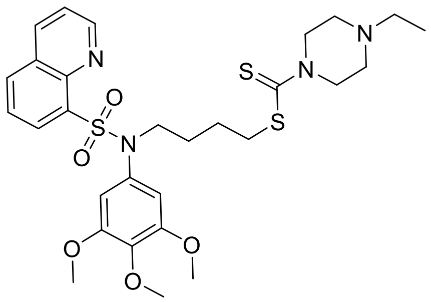

White solid, yield:85%, m.p.:110~112 °C. ^{1}H NMR (400 MHz, $CDCl_3$) δ 9.08 (dd, *J* = 4.2, 1.8 Hz, 1H), 8.28 – 8.09 (m, 2H), 7.92 (dd, *J* = 8.2, 1.3 Hz, 1H), 7.61 – 7.33 (m, 2H), 6.09 (s, 2H), 4.28 (s, 2H), 4.10 (t, *J* = 7.0 Hz, 2H), 3.90 (s, 2H), 3.68 (s, 3H), 3.43 (s, 6H), 3.26 (t, *J* = 7.4 Hz, 2H), 2.60 – 2.42 (m, 4H), 2.39 (q, *J* = 7.2 Hz, 2H), 1.79 (dd, *J* = 14.9, 8.0 Hz, 2H), 1.67 – 1.59 (m, 2H), 1.04 (t, *J* = 7.2 Hz, 3H). ^{13}C NMR (100 MHz, $CDCl_3$) δ 195.96, 151.90, 150.12, 143.25, 136.45, 136.14, 135.46, 133.62, 132.95, 132.25, 127.64, 124.58, 120.97, 105.52, 59.75, 54.88, 52.07, 51.15, 50.89, 35.65, 27.66, 25.04, 10.96. HRMS (m/z): Calcd. $C_{29}H_{39}N_4O_5S_3$, $[M+H]^+$ m/z: 619.2083, found: 619.2087.

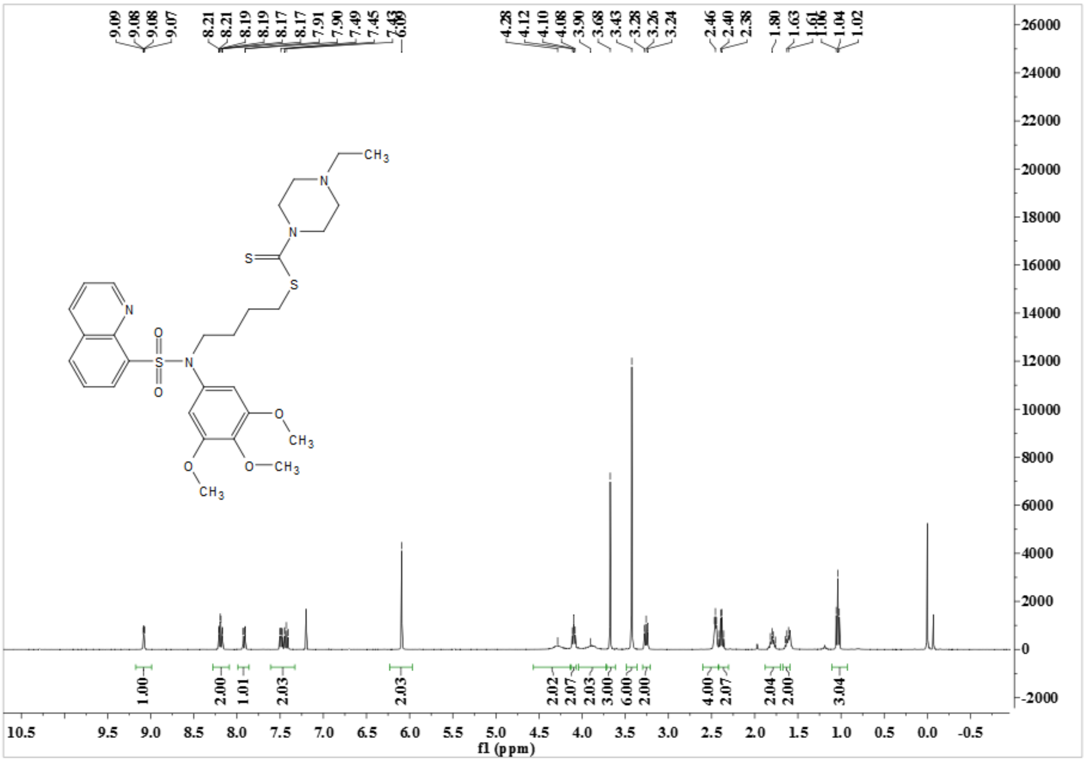

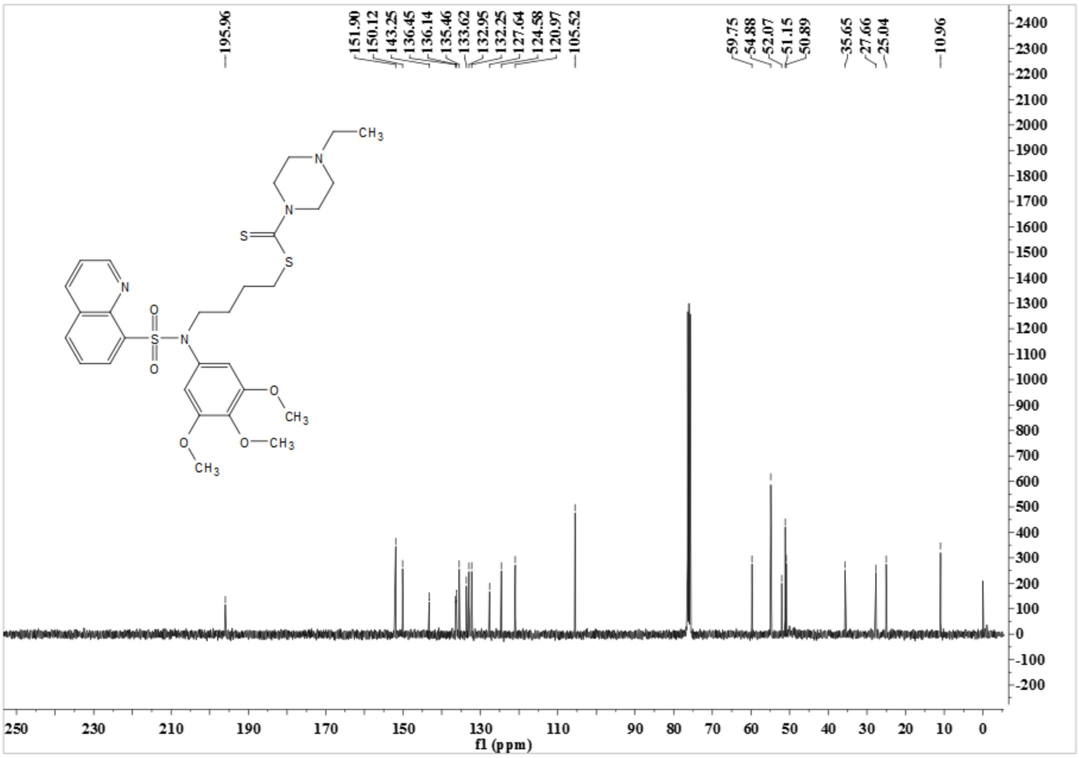

Tert-butyl-4-(((4-(N-(3,4,5-trimethoxyphenyl)quinoline-8-sulfonamido)butyl)thio)carbonothioyl)piperazine-1-carboxylate (L6)

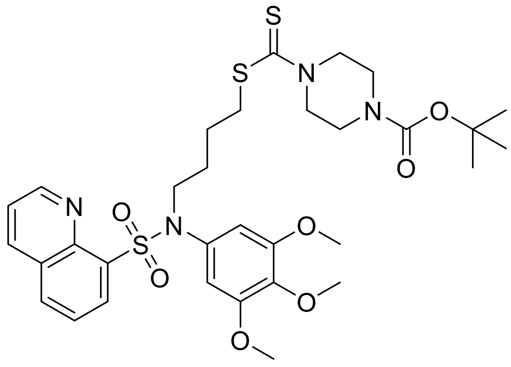

White solid, yield:74%, m.p.:98~100 °C. ^{1}H NMR (400 MHz, $CDCl_3$) δ 9.08 (dd, *J* = 4.2, 1.7 Hz, 1H), 8.31 – 8.13 (m, 2H), 7.92 (dd, *J* = 8.2, 1.2 Hz, 1H), 7.59 – 7.38 (m, 2H), 6.09 (s, 2H), 4.10 (t, *J* = 7.0 Hz, 6H), 3.67 (s, 3H), 3.54 – 3.46 (m, 4H), 3.42 (s, 6H), 3.27 (t, *J* = 7.4 Hz, 2H), 1.86 – 1.75 (m, 2H), 1.66 – 1.58 (m, 2H), 1.41 (s, 9H). ^{13}C NMR (100 MHz, $CDCl_3$) δ 157.80, 153.47, 151.90, 150.11, 143.23, 136.46, 136.09, 135.47, 133.59, 132.97, 132.27, 127.65, 124.61, 120.97, 105.49, 79.55 , 59.75, 54.87, 52.07, 35.65, 27.62, 27.35, 24.95. HRMS (m/z): Calcd. $C_{32}H_{43}N_4O_7S_3$, $[M+H]^+$ m/z: 691.2294, found: 691.2298.

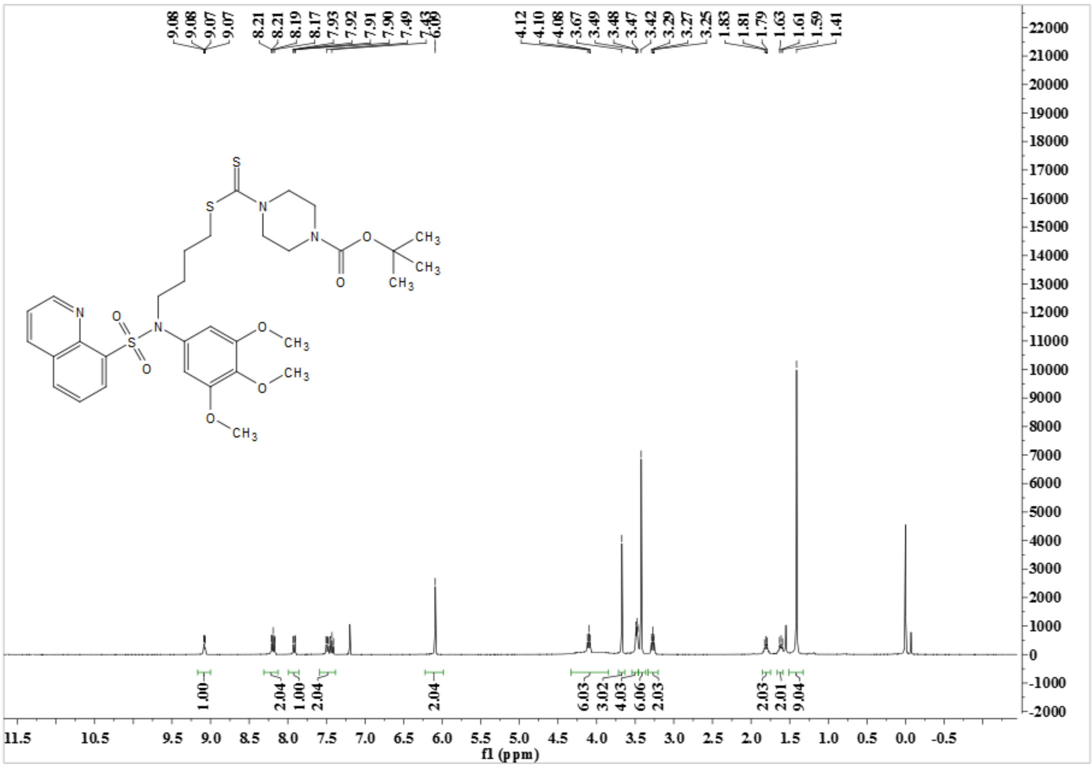

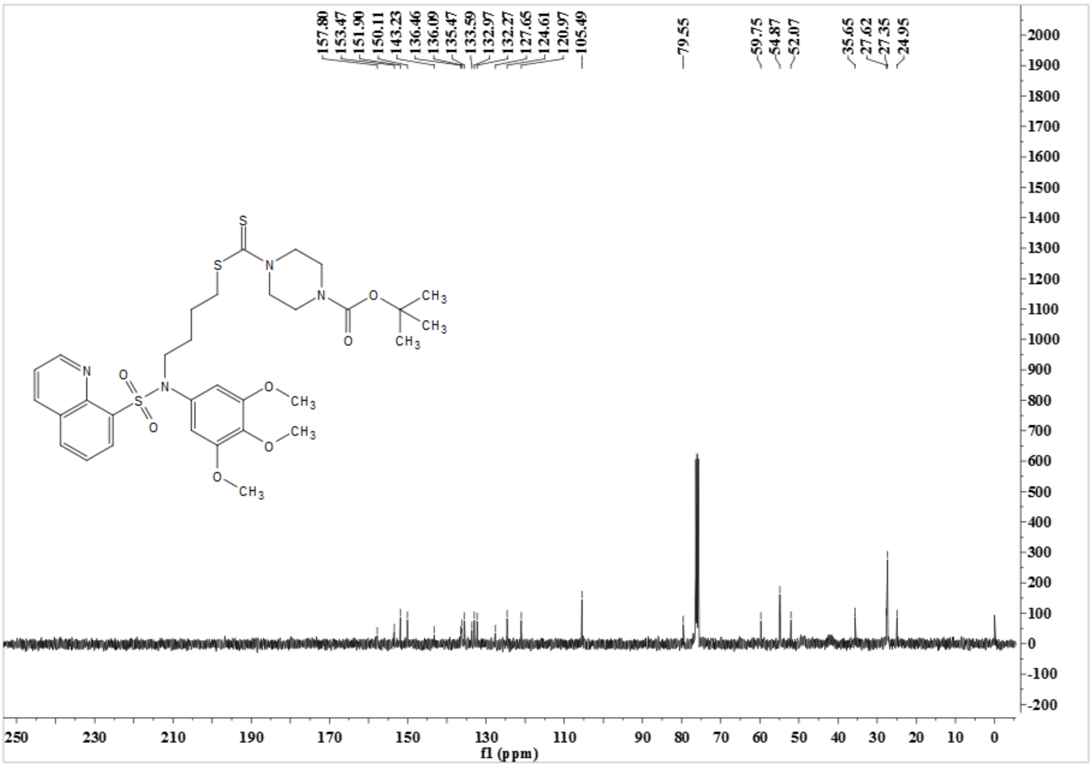

4-(N-(3,4,5-trimethoxyphenyl)quinoline-8-sulfonamido)butyl-4-methylpiperazine-1-carbodithioate (L7)

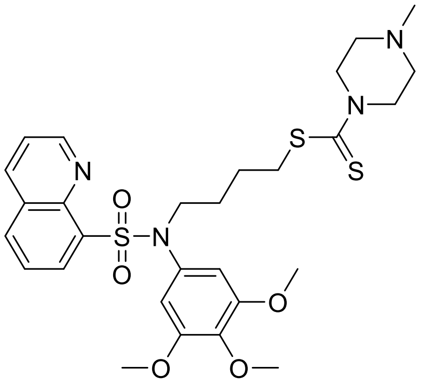

White solid, yield:69%, m.p.:135~137 °C. ^{1}H NMR (400 MHz, DMSO) δ 9.17 (dd, *J* = 4.2, 1.7 Hz, 1H), 8.56 (dd, *J* = 8.4, 1.6 Hz, 1H), 8.23 (ddd, *J* = 42.3, 7.8, 1.2 Hz, 2H), 7.78 – 7.59 (m, 2H), 6.17 (s, 2H), 4.23 (s, 2H), 4.08 (t, *J* = 6.7 Hz, 2H), 3.88 (s, 2H), 3.56 (s, 3H), 3.41 (s, 6H), 3.24 (t, *J* = 7.3 Hz, 2H), 2.44 – 2.29 (m, 4H), 2.21 (s, 3H), 1.72 (dd, *J* = 14.7, 7.7 Hz, 2H), 1.52 (dd, *J* = 14.5, 6.9 Hz, 2H). ^{13}C NMR (100 MHz, DMSO-d_6) δ 195.24, 152.43, 151.46, 143.31, 136.92, 136.71, 136.27, 134.27, 134.04, 133.23, 128.48, 125.62, 122.49, 106.15, 59.93, 55.52, 53.94, 52.16, 45.05, 35.70, 27.83, 25.74. HRMS (m/z): Calcd. $C_{28}H_{37}N_4O_5S_3$, $[M+H]^+$ m/z: 605.1926, found: 605.1929.

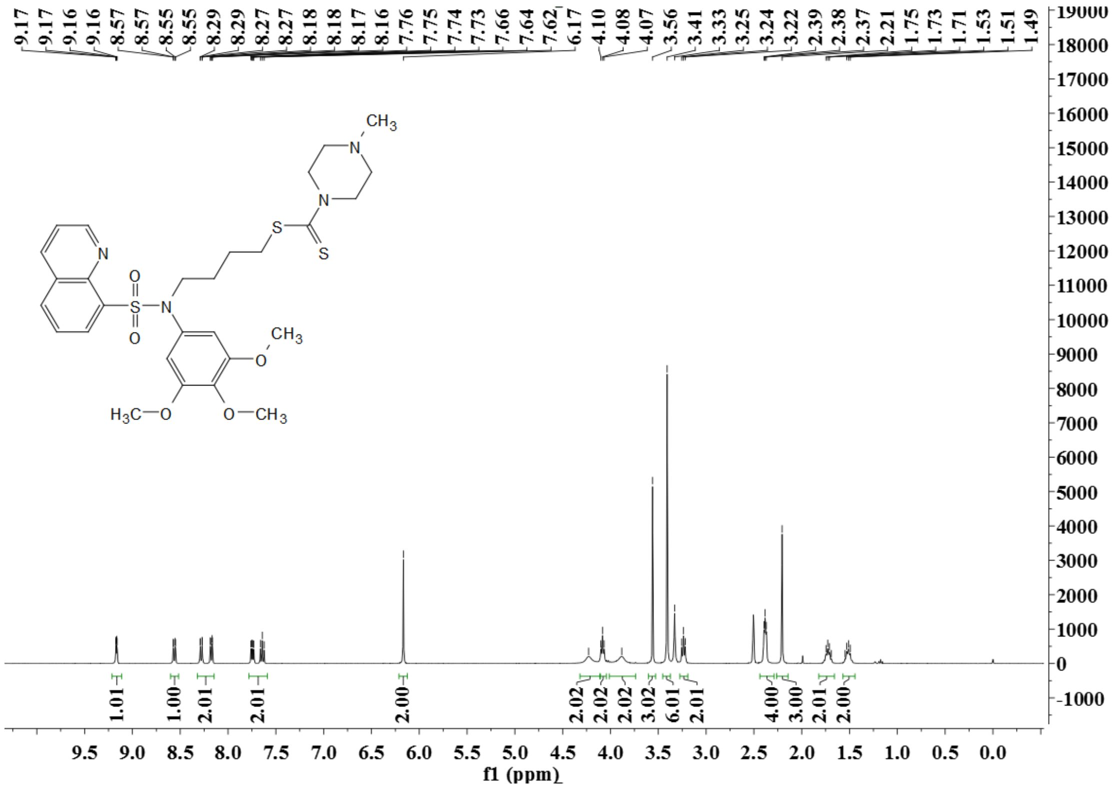

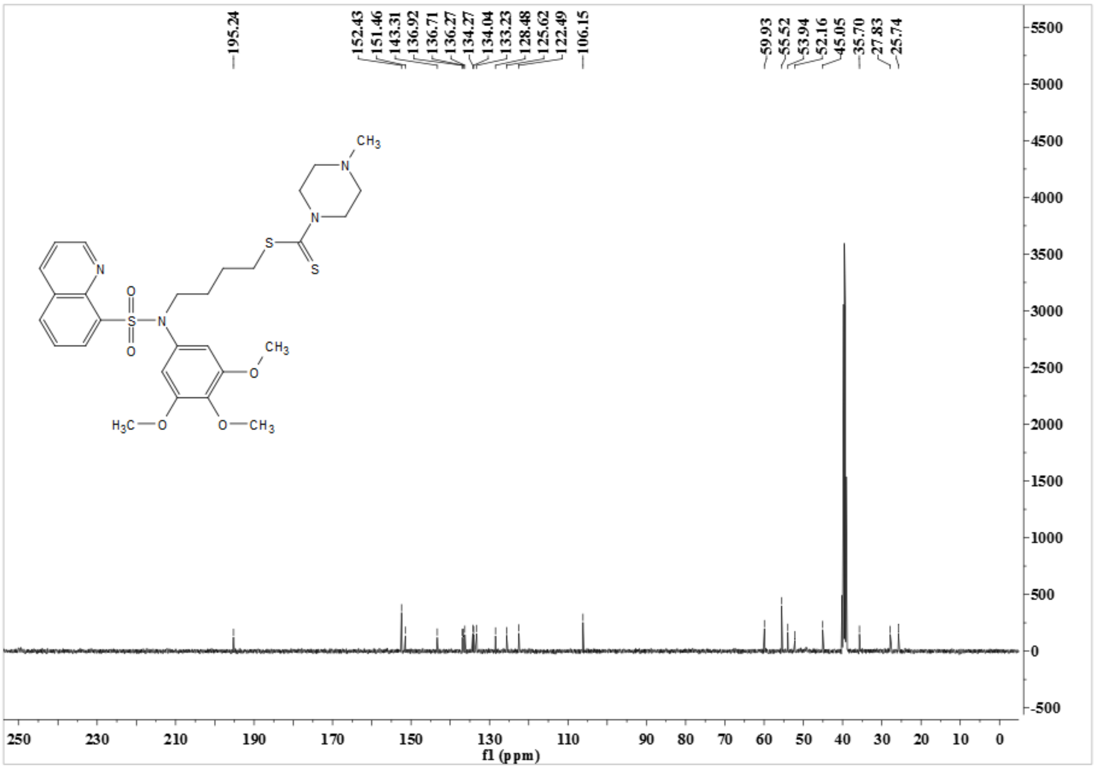

4-(N-(3,4,5-trimethoxyphenyl)quinoline-8-sulfonamido)butyl-4-(2-hydroxyethyl)piperazine-1-carbodithioate (L8)

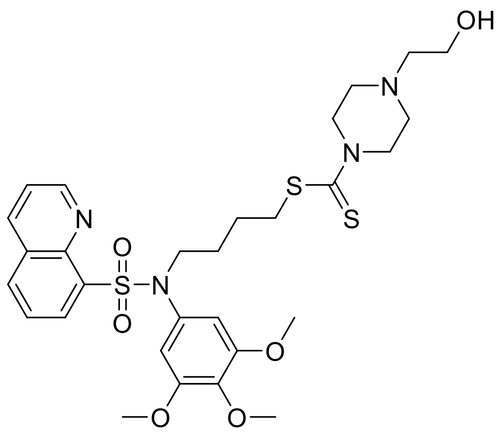

White solid, yield:73%, m.p.:94~96 °C. ^{1}H NMR (400 MHz, $CDCl_3$) δ 9.08 (dd, *J* = 4.1, 1.6 Hz, 1H), 8.19 (dd, *J* = 10.7, 3.9 Hz, 2H), 7.92 (d, *J* = 7.3 Hz, 1H), 7.53 – 7.40 (m, 2H), 6.09 (s, 2H), 4.28 (s, 2H), 4.10 (t, *J* = 7.0 Hz, 2H), 3.88 (d, *J* = 20.8 Hz, 2H), 3.67 (s, 3H), 3.64 – 3.57 (m, 2H), 3.42 (s, 6H), 3.26 (t, *J* = 7.4 Hz, 2H), 2.53 (dd, *J* = 6.2, 3.8 Hz, 6H), 1.80 (dd, *J* = 14.8, 7.7 Hz, 2H), 1.65 – 1.55 (m, 2H). ^{13}C NMR (100 MHz, $CDCl_3$) δ 196.27, 151.91, 150.12, 143.25, 136.45, 136.13, 135.47, 133.61, 132.97, 132.25, 127.64, 124.60, 120.97, 105.52, 59.76, 58.04, 56.96, 54.88, 52.08, 51.32, 35.69, 27.63, 25.01. HRMS (m/z): Calcd. $C_{29}H_{39}N_4O_6S_3$, $[M+H]^+$ m/z: 635.2032, found: 635.2037.

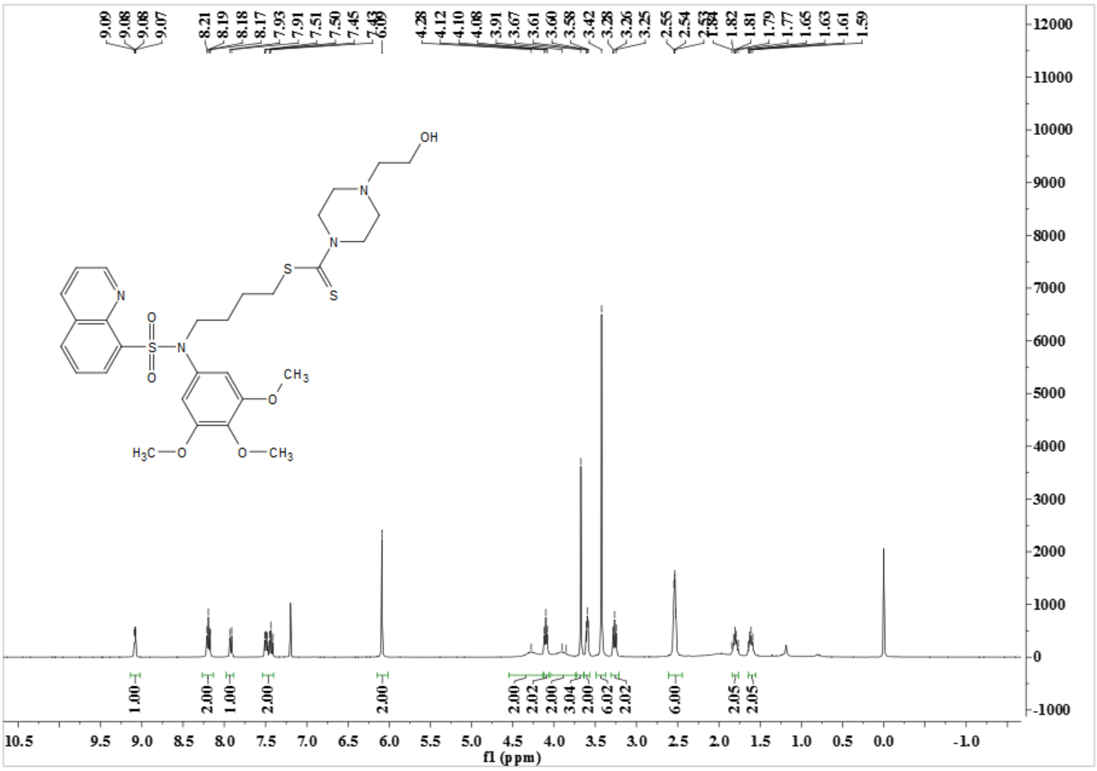

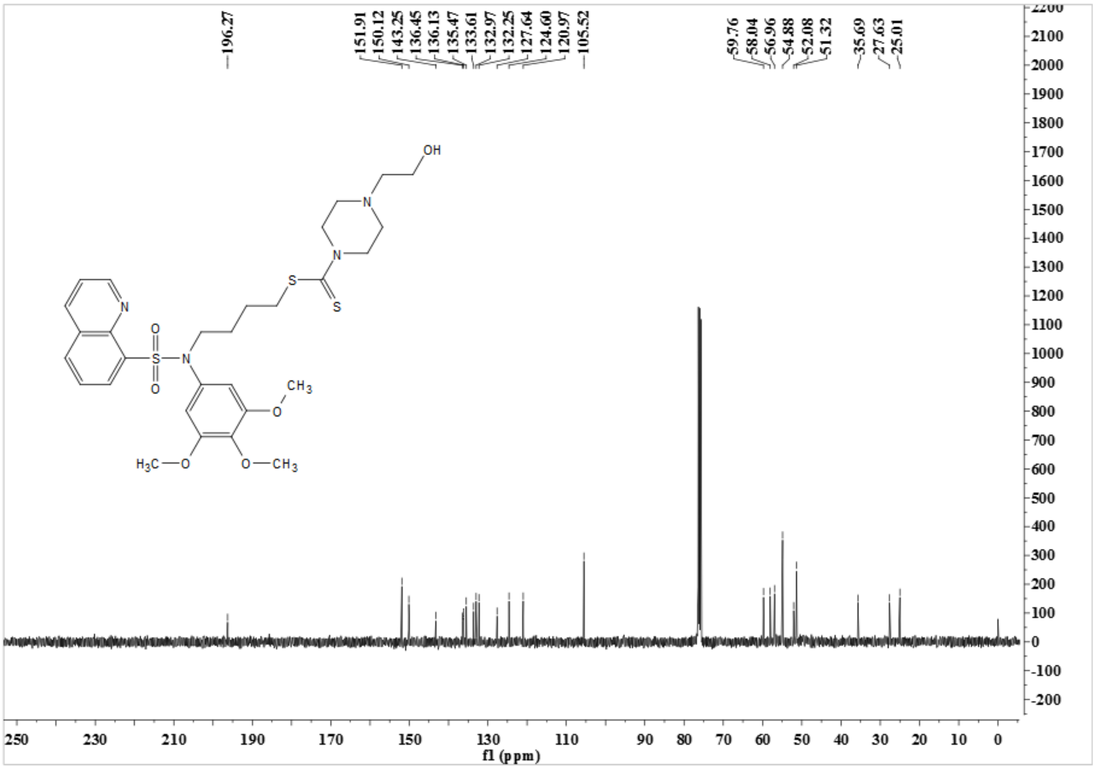

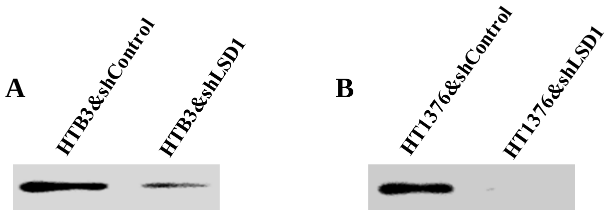

Figure 1S. (A) The expression levels of LSD1 in HTB3&shControl cells and HTB3&shLSD1 cells. (B) The expression levels of LSD1 in HT1376&shControl cells and HT1376&shLSD1 cells.
